# Synthesis of novel, DNA binding heterocyclic dehydroabietylamine derivatives as potential antiproliferative and apoptosis-inducing agents

**DOI:** 10.1080/10717544.2020.1716879

**Published:** 2020-01-27

**Authors:** Fengyi Zhao, Xu Sun, Wen Lu, Li Xu, Jiuzhou Shi, Shilong Yang, Mengyi Zhou, Fan Su, Feng Lin, Fuliang Cao

**Affiliations:** a Co-Innovation Center for Sustainable Forestry in Southern China, Nanjing Forestry University, Nanjing, PR China;; b College of Forestry, Nanjing Forestry University, Nanjing, PR China;; c College of Science, Nanjing Forestry University, Nanjing, PR China;; d College of Information Science and Technology, Nanjing Forestry University, Nanjing, PR China;; e Advanced Analysis and Testing Center, Nanjing Forestry University, Nanjing, PR China

**Keywords:** Dehydroabietylamine derivatives, antiproliferative, lower toxicity, apoptosis

## Abstract

Several dehydroabietylamine derivatives containing heterocyclic moieties such as thiophene and pyrazine ring were successfully synthesized. The antiproliferative activities of these thiophene-based Schiff-bases, thiophene amides, and pyrazine amides were investigated *in vitro* against Hela (cervix), MCF-7 (breast), A549 (lung), HepG2 (liver), and HUVEC (umbilical vein) cells by MTT assay. The toxicity of **L^1^**−**L^10^**(IC_50_ = 5.92− >100 μM) was lower than **L^0^** (1.27 μM) and DOX (4.40 μM) in every case. Compound **L^1^** had higher anti-HepG2 **(**0.66 μM), anti-MCF-7 (5.33 μM), and anti-A549 (2.11 μM) and compound **L^3^** had higher anti-HepG2 (1.63 μM) and anti-MCF-7 (2.65 μM) activities. Both of these compounds were recognized with high efficiency in apoptosis induction in HepG2 cells and intercalated binding modes with DNA. Moreover, with average IC_50_ values of 0.66 and 5.98 μM, **L^1^** was nine times more effective at suppressing cultured HepG2 cells viability than normal cells (SI = 9). The relative tumor proliferation rate (T/C) was 38.6%, the tumor inhibition rate was up to 61.2%, which indicated that **L^1^** had no significant toxicity but high anti-HepG2 activity *in vivo*. Thus, it may be a potential antiproliferation drug with nontoxic side effects.

## 1. Introduction

Currently, various chemotherapy drugs have been developed for the treatment of different cancers; however, undesirable side effects may greatly impede their use in clinical progression. Among a variety of cancer cures, the antiproliferative approach plays a crucial role in controlling this deadly disease (Xin et al., 2018; Lei et al., 2019b). Up to now, numerous molecules containing heterocyclic rings have showed great antiproliferative potential, particularly those with thiophene and pyrazine rings.

Thiophene derivatives are ubiquitous in nature and can be found in the structure of various drugs and medicines, produced by combustion of fossil fuels or by the general cooking process (Dyreborg et al., 1996; Dalvie et al., 2002; Medower et al., 2008). Thiophenes bear extensive pharmacological properties such as analgesic, antipyretic, and antiandrogenic activities (Hana et al., 2008; Huang et al., 2012; Iványi et al., 2012). Furthermore, a great number of thiophene derivatives are used as antitumor agents (Lesyk et al., 2006; Kulandasamy et al., 2009; Khalil et al., 2010; Ye et al., 2010). For instance, OSI-930 is an investigational anticancer agent which contains thiophene moiety (Petti et al., 2005; Garton et al., 2006). Ghorab et al. (2016) reported a series of thiophene derivatives with high anti-MCF-7 (breast adenocarcinoma) cancer activity (half maximal inhibitory concentration [IC_50_] = 33.1–66.3 μM). Mohareb and Al-Omran (2012) have studied three thiophene derivatives exhibited much higher inhibitory effects toward three tumor cell lines, MCF-7, NCI-H460 (non-small cell lung cancer), and SF-268 (CNS cancer) with GC_50_ value in the range of 0.01–16.2 μM than the reference drug, doxorubicin (DOX).

Recently, nitrogen heterocycles have also been reported to exhibit therapeutics anticancer (Azuine et al., 2004; Lei et al., 2019a,c) and anti-microbial (Nomiya et al., 2000; Mathew et al., 2006) activities. Among them, the pyrazine heterocycles have widespread application in food science, materials, and medicinal chemistry (Mondal et al., 2010; Saito et al., 2010; Badrinarayanan & Sperry, 2011; Zitko et al., 2011). For example, pyrazinamide, a pyrazine derivative, is an antimicrobial agent that is most commonly used for treatment of active tuberculosis during the initial phase of therapy in combination with other agents. Quinoxaline compounds have been reported to possess a wide range of interesting biological properties such as anticancer, antiviral, antimicrobial, antifungal, antitubercular, anti-inflammatory, and anti-angiogenesis agents (Seitz et al., 2002; Smits et al., 2008; Vicente et al., 2009; Lee et al., 2010; Sridevi et al., 2010; Ingle et al., 2013; Aissi et al., 2014; Soozani et al., 2018), containing pyrazine motif. Ahmed et al. (2018) have synthesized several compounds and evaluated anticancer effects against three cancer lines (HCT-116, MCF-7, and HepG2), and the results revealed that pyrazine derivatives were the most active compounds with IC_50_ value of 1.89 and 2.05 Μm. Another pyrazine derivative, pyrazin-2(1H)-one, has attracted considerable attention due to its biological activities, such as anti-viral, antibacterial, anti-inflammatory, and anticancer (colon cancer therapies) activities (Lindsley et al., 2005).

Recently, dehydroabietylamine (**L^0^**), which is one of the most vital modified products of rosin, has attracted considerable attention due to the broad spectrum of biological properties (Singh et al., 2014; Lin et al., 2015; Auxiliadora et al., 2016; Bahekar et al., 2016; Fei et al., 2016; Liu et al., 2016; Wang et al., 2016; Huang et al., 2017; Liu et al., 2017). In general, a focus of research on dehydroabietylamine derivatives with their anticancer, antibacterial, antifungal, and cytotoxic activities has been paid their attention in forest chemistry too. Rao et al. (2012) screened a series of imines, amides, and ureas with a dehydroabietyl skeleton for their anticancer activities against SMMC7721 (liver), A549 (lung), C6 (glioma), and MCF-7 cancer cell lines with smallest IC_50_ values of 6.65, 0.75, 0.81, and 10.65 μM. Lately, our group has found that several Schiff-bases and amide compounds displayed highly potent inhibitory activities against HepG2 (liver), MCF-7 and A549 cells with smallest IC_50_ values of 0.14, 0.24, 2.58, and 3.17 μM (Zhao et al., 2018).

Up until this day, finding new molecules with more effective, less toxic, and target-specific DNA binding properties is one of the most important interest in medicinal chemistry. Cisplatin, a well-known active anticancer drug, can covalently bind to DNA, but its usage is limited with side effects and acquired cellular resistance (Rajendiran et al., 2007). The aforementioned thiophene, pyrazine, and dehydroabietylamine analogs are all recognized as excellent antiproliferative agents; thus, our group was interested in the further investigation of these derivatives. Our goal was to achieve high anticancer activity, low toxicity, and target-specific DNA binding properties with the dehydroabietylamine derivatives including thiophene Schiff-bases (**L^1^−L^3^**), thiophene amides (**L^4^−L^6^**), and pyrazine amides (**L^7^−L^10^**). These new compounds were screened for antiproliferative activities against Hela (cervix), MCF-7, A549, HepG2 human cancer cell lines *in vitro* by MTT assay, in addition to **L^1^** *in vivo*. We have verified that several compounds owned high antiproliferative activities against these cancer cells and some of them exhibited more potent antiproliferative activities as compared to dehydroabietylamine. Subsequently, the induction of apoptosis effect on **L^1^**and **L^3^** with HepG2 cells was also investigated and the result suggested they could inhibit cell proliferation by inducing apoptosis. In this report, we hope to display a simple but effective strategy that may make contributions to the exploration of future anticancer drugs.

## 2. Results and discussion

### 2.1. Chemistry

**Scheme 1.**
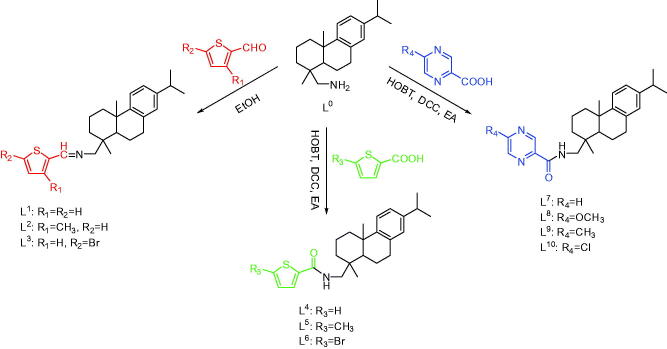
Synthesis of **L^1^−L^10^**.

All compounds were synthesized by the facile and efficient synthetic route from the commercially available (+)-Dehydroabietylamine, [α]20 D = +55.1 (c 2.4 pyridine) (Scheme 1). To explore the relationship between the compound’s structures and biological activities, three thiophene aldehydes with different functional groups, three carboxylic acids, and four pyrazine carboxylic acids with various substituents were designed to react with dehydroabietylamine to prepare thiophene Schiff-bases (**L^1^−L^3^**), thiophene amides (**L^4^−L^6^**), and pyrazine amides (**L^7^−L^10^**). **L^1^−L^10^** were obtained under neutral to slightly alkaline conditions (pH = 7.0–7.4) during the experimental process.

**Figure 1.**
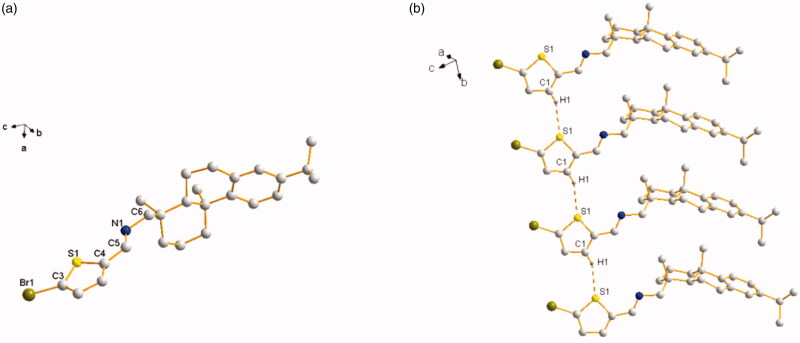
(a) Molecular structure of **L^3^** (hydrogen atoms omitted for clarity). (b) The 1D chain structure formed by intermolecular hydrogen bonds.

**Figure 2.**
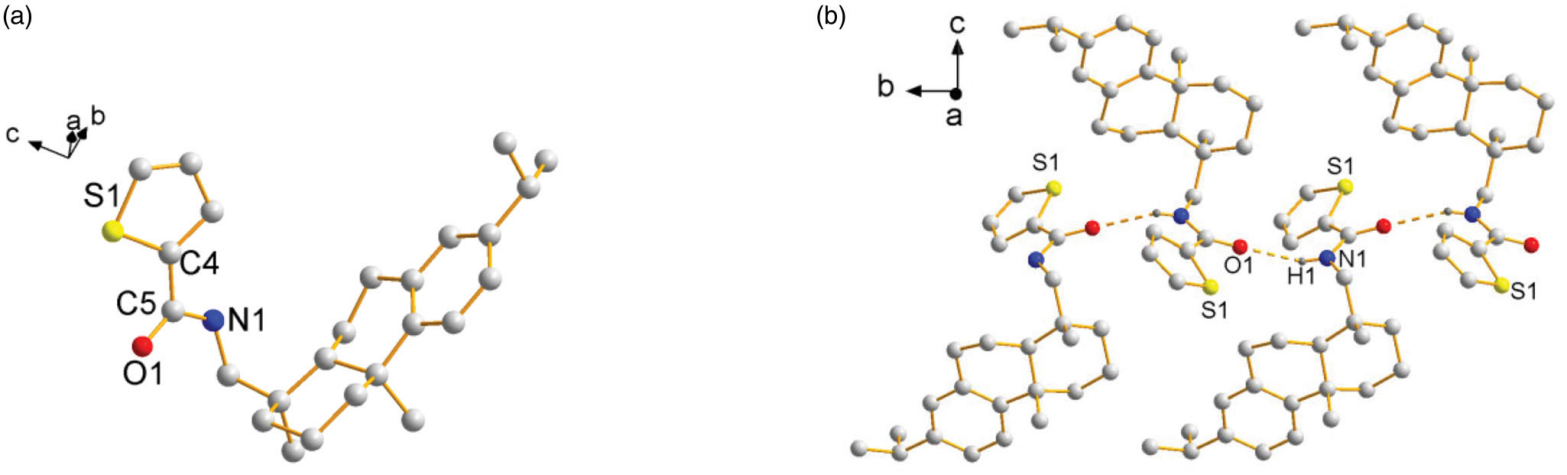
(a) Molecular structure of **L^4^** (hydrogen atoms omitted for clarity). (b) The 1D chain structure formed by intermolecular hydrogen bonds.

Both of the thiophene Schiff-bases **L^3^** and thiophene amides **L^4^**owned novel structural features with two aromatic rings (thiophene ring and benzene ring) and two aliphatic rings from dehydroabietylamine. For **L^3^**, its faint yellow block-shaped single crystal was found to be a monoclinic crystal in a chiral space group *P*2_1_ with a Flack parameter of 0.009(8). The thiophene ring with C5, N1, and C6 was coplanar (Figure 1(a)). The molecules are stably connected by slightly weak C1 − H1⋅⋅⋅S1^#^ hydrogen bond to assemble an infinite one-dimensional chain structure along *b* axis (Figure 1(b)). The distance of H1⋅⋅⋅S1 (2.8019(18) Å) and C1⋅⋅⋅S1 (3.6459(64) Å) is corresponding to Shi’s report (H16⋅⋅⋅S2 2.95 Å and C16⋅⋅⋅S2 3.61 Å), which is slightly shorter than the sum of van der Waals radii, proving that a weak interaction existed between H1⋅⋅⋅S1 (Shi & Wen, 1998). The length of the new imine double bond C5 − N1 (1.2520(7) Å) is in accordance with the report from Lu (C10 − N2 1.2913(3) Å) (Lu et al., 2016) and our previous result of C7 − N1 1.2690(4) Å (Zhao et al., 2018). For **L^4^**, its colorless block-shaped single crystal was obtained as orthorhombic crystal system in a chiral space group *P*2_1_ with a Flack parameter of 0.08(5). The molecules are connected by N1 − H1⋅⋅⋅O1^#^ hydrogen bond to assemble an infinite one-dimensional chain structure along *b* axis (Figure 2(b)). The hydrogen-bond parameters of **L^3^** and **L^4^** are shown in Table 1. Selected bond lengths and angles of **L^3^** and **L^4^**are shown in Table 2. The crystallographic data are shown in Table S1 in Supplementary Material.

**Table 1.** Hydrogen bonds of **L^3^** and **L^4^**.

Compd.	D–H⋅⋅⋅A	d(D–H)(Å)	d(H⋅⋅⋅A)(Å)	d(D⋅⋅⋅A)(Å)	∠DHA( ° )
**L^3^**	**C1–H1⋅⋅⋅S1**^a^	0.9304(62)	2.8019(18)	3.6459(64)	151.365(379)
**L^4^**	**N1–H1⋅⋅⋅O1**^b^	0.8579(30)	2.2648(30)	3.0015(42)	143.759(209)

Symmetry code: ^a^ *x*, –2 + *y*, *z*; ^b^ *x*, –1 + *y*, *z*.

**Table 2.** Selected bond lengths (Å) and angles (deg) for **L^3^** and **L^4^**.

L^3^				L^4^	
Br1 − C3	1.866(7)	N1–C6	1.471(7)	C5 − O1	1.226(5)
S1 − C4	1.706(6)	C4 − S1 − C3	91.6(3)	N1 − C5	1.346(5)
S1 − C3	1.709(6)	C5 − N1 − C6	115.7(5)	O1 − C5 − N1	123.7(4)
N1 − C5	1.252(7)	S1 − C3 − Br1	120.1(4)	C1 − S1 − C4	91.3(2)

### 2.2. Biological evaluation

#### 2.2.1. Antiproliferative activities

Compounds **L^1^**−**L^10^** were evaluated against human cancer cell lines Hela, HepG2, MCF-7, A549, and the normal cell HUVEC with the antiproliferative activities *in vitro* by MTT assay, and results are summarized as IC_50_ values in Table 3. DMSO was used as negative control and DOX (Doxorubicin) as positive control, which is a common chemotherapy medication used to cure cancer (Wang et al., 2004).

**Table 3.** Cytotoxicity of **L^1^**−**L^10^** and DOX against certain axenic cancer cells and normal cell.

**IC_50_ ± SE**^a^**( *μ*M )**					
Compd.	Hela	HepG2	MCF-7	A549	HUVEC
**L^0^**	2.02 ± 0.02	2.56 ± 0.04	19.45 ± 0.39	5.02 ± 0.19	1.27 ± 0.03
**L^1^**	9.02 ± 0.25***	0.66 ± 0.02***	5.33 ± 0.16***	2.11 ± 0.06***	5.92 ± 0.17***
**L^2^**	59.52 ± 0.27***	28.78 ± 0.19***	14.48 ± 2.48*	>100	34.02 ± 1.57***
**L^3^**	54.38 ± 0.38***	1.63 ± 0.39***	2.65 ± 0.07***	95.48 ± 2.48***	16.65 ± 1.08*
**L^4^**	14.80 ± 0.22^ns^	45.99 ± 1.13^ns^	8.21 ± 0.65***	>100	>100
**L^5^**	>100	53.14 ± 1.95***	8.27 ± 0.21***	>100	>100
**L^6^**	>100	51.44 ± 2***	55.56 ± 2.56***	94.39 ± 2.56***	75.45 ± 12.44***
**L^7^**	43.53 ± 1.34***	24.01 ± 1.33***	11.82 ± 0.54**	83.60 ± 15.65***	66.77 ± 2.60***
**L^8^**	20.22 ± 0.89**	17.06 ± 0.07**	8.81 ± 0.54***	36.02 ± 2.18**	95.11 ± 0.97***
**L^9^**	52.46 ± 0.54***	44.94 ± 0.75***	6.66 ± 0.24***	>100	18.91 ± 1.72**
**L^10^**	>100	56.08 ± 6.11***	88.81 ± 3.46***	39.63 ± 1.74**	29.38 ± 2.35***
**DOX**	3.55 ± 0.17^ns^	1.20 ± 0.04^ns^	14 ± 1.03*	3.35 ± 0.69^ns^	4.40 ± 0.55***

ns: not significant, complexes compared with **L^0^**, respectively.

^a^Average IC_50_ values from at least three independent experiments.

***p < .001.

**p < .01.

*p < .05.

**Figure 3.**
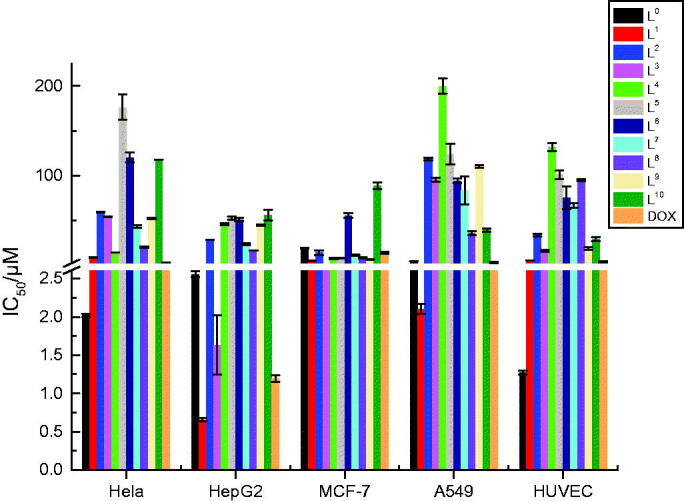
The comparison of antiproliferative effects and cytotoxicity of DOX, **L^0^**−**L^10^**.

A histogram was drawn more distinctly to compare the antiproliferative activity of **L^0^−L^10^** against axenic cancer cells and cytotoxicity based on IC_50_ values (Table 3). We used DOX as positive control, **L^0^** also as control to compare the antiproliferative activity among its modified products for the reasoned that the antiproliferative activity of **L^0^** was slightly lower than DOX (except Hela cells), whereas that was better than a variety of pharmaceutical products. The values of *p* > 0.05 were considered that the antiproliferative activity of **L^0^** and DOX in statistics difference were insignificant, except for MCF-7 cells. The result is shown in Figure 3.

From Figure 3, the result showed quite clearly that **L^1^** and **L^3^** had higher antiproliferative activity against HepG2 cells while **L^2^** had higher antiproliferative activity against A549 cells. **L^1^**−**L^10^** were low toxic as their toxicity (IC_50_ = 5.92− >100 μM) was all lower than **L^0^** (1.27 μM) and DOX (4.40 μM) for HUVEC cells. For further observation, for HepG2 cells, the IC_50_ values of **L^1^** (0.66 μM) and **L^3^** (1.63 μM) were much smaller than those of **L^0^**. Moreover, with average IC_50_ values of 0.66 and 5.92 μM, **L^1^** was nine times more effective at suppressing cultured HepG2 cells viability than normal cells, and **L^3^** as well with average IC_50_ values of 1.63 and 16.65 μM. For MCF-7 cells, most compounds had higher anti-MCF-7 activity than both **L^0^** and DOX with their smaller IC_50_ value compared to **L^0^** (excluding **L^6^** and **L^10^**), particularly, **L^1^** (5.33 μM) and **L^3^** (2.65 μM). For A549 cells, the IC_50_ value of **L^1^** (2.11 μM) was smaller than **L^0^**, which meant **L^1^** had higher anti-A549 activity than **L^0^**; in especial, the IC_50_ value of **L^1^** was smaller than DOX. The security index (SI) value of **L^1^** is 9.0 and **L^3^** is 10.2, which suggested they had higher anticancer activity and lower toxicity compared with L**^0^（**0.5）and DOX (3.7). Actually, among these investigated compounds, **L^1^** had the smallest IC_50_ value of 0.66 μM against HepG2 cells and lower toxicity, which shows that **L^1^** may be a promising anticancer drug.

Furthermore, by contrast with the structure and cytotoxicity *in vitro* on the cancer cell lines of **L^1^−L^10^**, compounds owned − CH_3_ electron-donating group (**L^2^**, **L^5^**, **L^9^**) had relatively lower antiproliferative activity than those without electron-donating group (**L^1^**, **L^4^**, **L^7^**). As reported by Hande, introduction of a polar group containing a hydrogen-bond donor resulted to enhanced anticancer activity (Hande, 1998). Although the correspondingly better solubility of Schiff-bases and the special structure of Schiff-base (−CH = N−), which can conjugate with the thiophene ring and grant it with higher stability and bioactivity, these Schiff-base compounds (**L^1^**−**L^3^**) had higher antiproliferative activity against HepG2 and MCF-7 cells. As a whole, all these synthetic compounds could inhibit the proliferation of cancer cells by penetrating into cytoplasm with endocytosis, we reasoned that the strong chelating ability of ‘S’ and ‘N’ may lead to the easy formation of hydrogen bonds with carboxyl and amino groups in the cancer cell lines through their unique lone pair electrons.

#### 2.2.2. Induction of apoptosis

**Figure 4.**
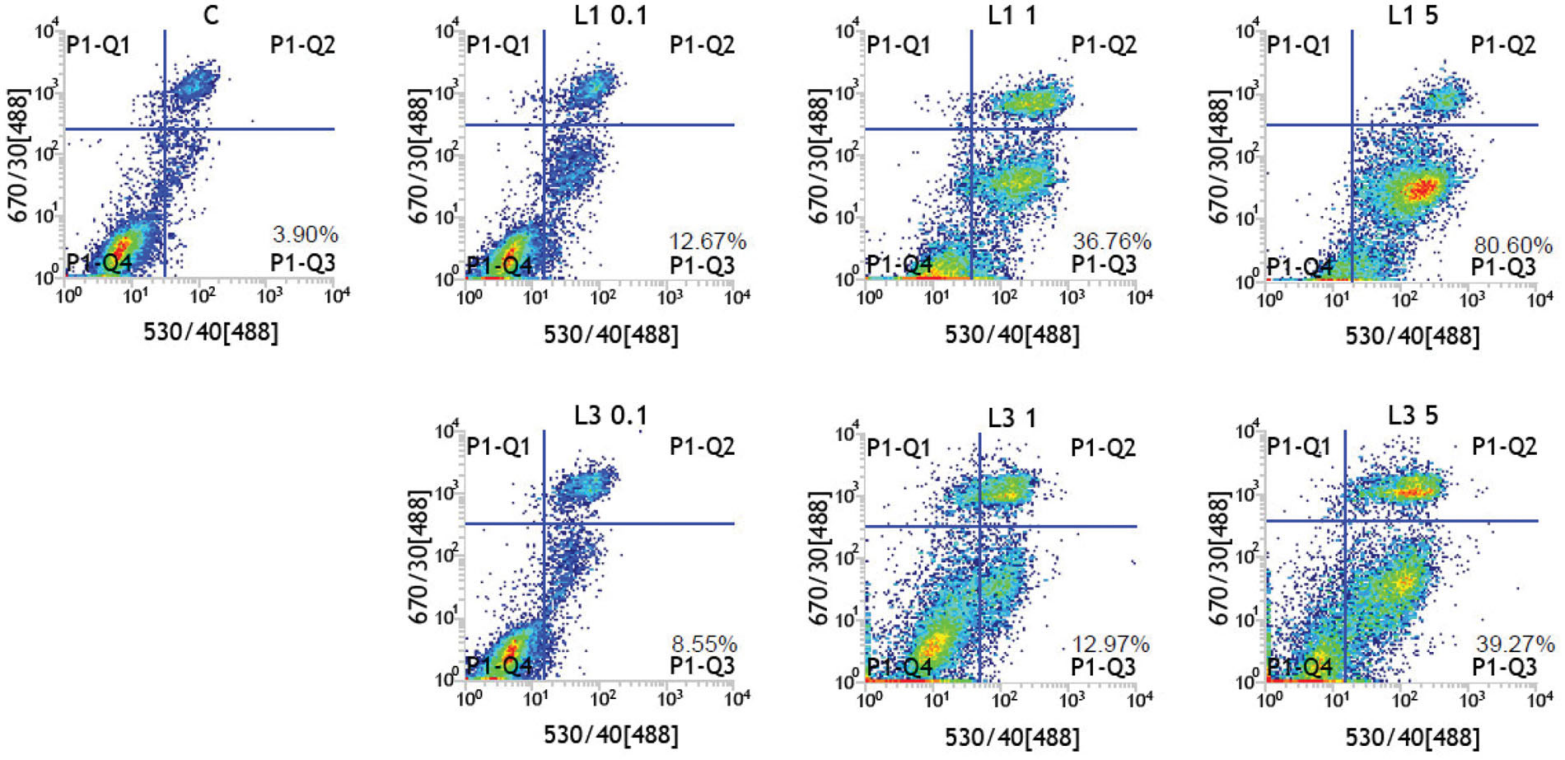
Apoptosis assay of HepG2 cells treated with **L^1^** and **L^3^**. C was DMSO (negative control), others were **L^1^** at 0.26, 2.6, 13 μM, and **L^3^** at 0.22, 2.2, 11 μM, respectively.

As a routine chemotherapeutic agent for a broad range of malignancies, DOX can prevent tumor proliferation via inducing apoptosis. Apoptosis, known as programed cell death, is a crucial process related to the regulation of development and homeostasis (Arjmand & Aziz, 2009; Qi et al., 2019). It plays an important role in cancer, as its induction in cancer cells is significant to a successful therapy, thereby carrying out apoptosis assay can afford meaningful information to the study of the mode of action. In this paper, we have estimated the potential mechanism of cell proliferation inhibitory activity of **L^1^** and **L^3^** through the assay apoptosis with Annexin V-FITC/PI and flow cytometry in HepG2 cells. The flow-cytometric analysis is shown in Figure 4.

According to Figure 4, after **L^1^** and **L^3^** incubated with HepG2 cells with the concentration range from 0.22 to 13 μM, respectively, live cells reduced and apoptotic cells increased; herein, they obviously exhibited the dosage-dependent manner. When the concentrations of **L^1^** were 0.26, 2.6, and 13 μM, the apoptotic ratios of **L^1^** were 12.67%, 36.76%, and 80.60%, respectively. Simultaneously, when the concentrations of **L^1^** were 0.22, 2.2, and 11 μM, the apoptotic ratios of **L^3^** were 8.55%, 12.97%, and 39.21%, respectively. Compared to the apoptotic ratio of these two compounds, **L^1^**had obviously higher ability to induce apoptosis at the similar concentration. During the induction of apoptosis process, live cells trend to develop toward apoptotic cells with the enhanced concentration. Herein the reported results distinctly illustrated that **L^1^** and **L^3^**could inhibit cell proliferation by inducing apoptosis.

For further investigation, the logP values of all compounds were calculated by hyperchem 8.0 (Table 4). Compared with DOX (1.50), the values of logP of **L^1^** (0.98), **L^2^** (1.17) and **L^3^** (1.32) were smaller with stronger hydrophily, which implied they had better solubility and may be in favor of excellent cytotoxic activities. According to a report, the ideal drug potency and satisfied pharmacokinetic profiles required good water solubility to achieve the desired therapeutic efficacy (Li et al., 2014). Any drug to be absorbed should exist in the form of solution at the site of absorption (Muhammad & Bashir, 2017). The value of logP of **L^4^**−**L^10^** (>3) was larger, which suggested they had stronger lipophicity and its slightly poor solubility might lead to relatively worse effects on cancer cells. All compounds herein had been next examined with p*K*_b_ in the range of 9.00–14.96; thus, they seemed no regular effect on cytotoxic activities.

**Table 4.** Physicochemical data (logP and p*K*_b_) of **L^1^−L^10^**, DOX.

Compd.	**L^0^**	**L^1^**	**L^2^**	**L^3^**	**L^4^**	**L^5^**	**L^6^**	**L^7^**	**L^8^**	**L^9^**	**L^10^**	**DOX**
**logP**	0.40	0.98	1.17	1.32	3.46	3.48	4.90	3.65	4.35	3.68	4.79	1.50
**p*K*_b_**	7.00	9.00	10.18	9.90	14.30	14.16	14.96	11.12	10.36	13.38	11.50	5.80

#### 2.2.3. DNA binding modes

**Figure 5.**
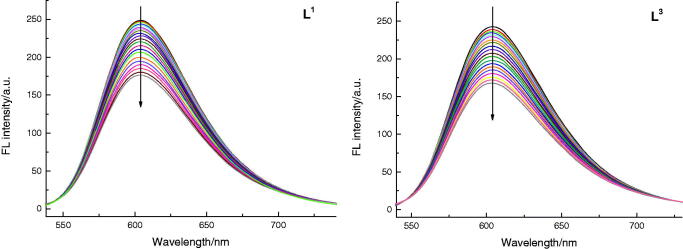
Emission spectra of DNA − EB in the absence and presence of increasing amounts of **L^1^** and **L^3^** at room temperature, respectively ([EB] = 2 × 10^−5^ M, [DNA] = 1 × 10^−4^ M, and [**L^1^**, **L^3^**] = 1.5 × 10^−5 ^M).

Intercalation is well known to strongly influence the properties of the DNA and has been reported as a preliminary step in mutagenesis. It was reported that DOX had the ability to remain inside nucleated cells because of its lipophilic characteristics and DNA intercalating or binding properties (Arjmand & Aziz, 2009; Sun et al., 2019). We further investigated whether **L^1^** and **L^3^** had similar activity to DOX; herein we studied DNA binding properties of them. The DNA (Salmon Sperm DNA) binding modes were evaluated by ethidium bromide (EB) fluorescence displacement experiments. Actually, EB had no perceptible emission in buffer solution; after adding DNA, the fluorescence intensity improved obviously, which was considered of its strongly intercalation with DNA base pairs. The intercalation of the compound with the base pairs of DNA can be confirmed when the DNA − EB emission can be decreased or quenched upon adding a compound (Li et al., 1996; Gao et al., 2010). As expected, the emission intensity apparently reduced (shown in Figure 5) by adding **L^1^** and **L^3^** to DNA-EB, which exhibits that **L^1^** and **L^3^** can bind to DNA at the sites occupied by EB, and it can interact with DNA by intercalation.

**Figure 6.**
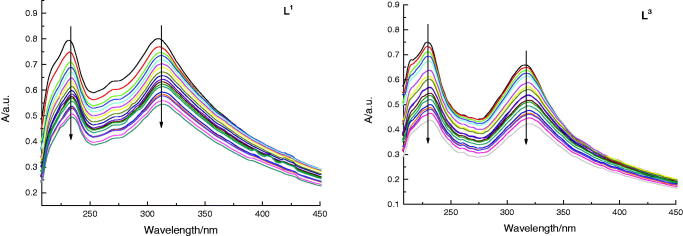
Absorption spectra of **L^1^** and **L^3^** (5 × 10^−5 ^M) in the absence and presence of increasing amounts of DNA (5 × 10^−5 ^M to 10^−3^ M) at room temperature in Tris-NaCl-HCl buffer (pH = 7.3). The arrow shows the absorbance change when increasing the DNA concentration.

The DNA binding modes of **L^1^** and **L^3^** were detected by the application of Absorption Spectral as well (shown in Figure 6). Normally, a compound binding to DNA can generate hypochromism and bathochromism by intercalation. The absorption spectra exhibited a hypochromic shift after promoting added amounts of DNA to solution of **L^3^**, which illustrated an intercalative binding mode. The result was in coincidence with that of fluorescence studies. To sum up, the absorption and fluorescence spectral all verified that **L^1^** and **L^3^** with the same as DOX could bind with DNA through intercalation.

In view of aforementioned findings, these synthetic dehydroabietylamine derivatives had relatively high potential antiproliferative activity and low toxicity, some of them also could induce apoptosis at low concentration. Moreover, to understand the cytotoxicity and the DNA binding modes was significative for designing new and potential drugs.

#### 2.2.4. Antiproliferation activity in vivo

Herein, we have intravenously injected compound **L^1^**(dose: 0.6 mg/kg) into the mice with HepG2 cells during 25 days with every 3 days *in vivo* experiment for further investigation.

**Figure 7.**
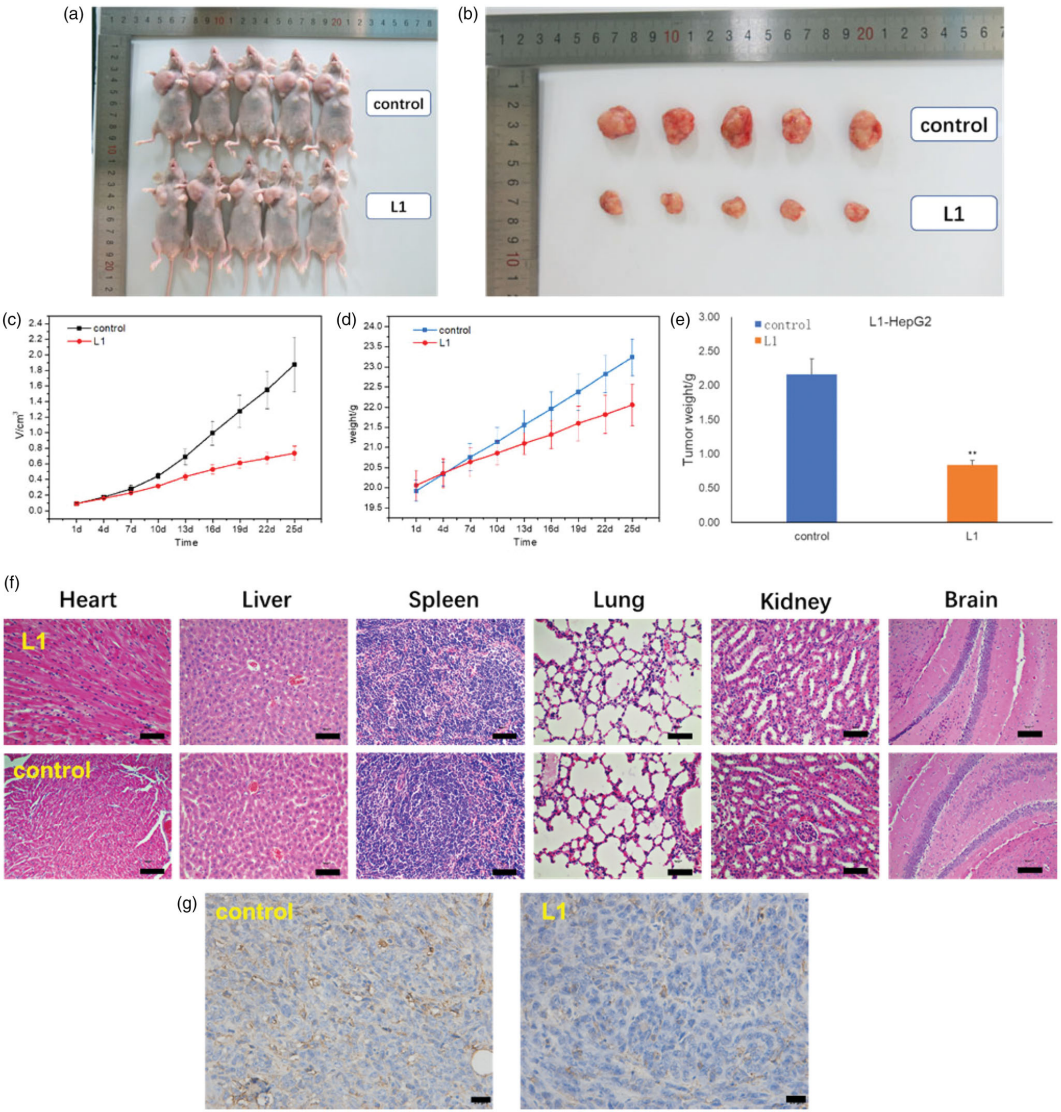
(a) Whole appearance and (b) the volume of tumor mice injected with PBS (control) and compound **L^1^** after 25 days. (c) Change in tumor volume of mice injected with compound **L^1^** (0.6 mg/kg) compared with PBS control. (d) Change in body weight of mice injected with compound **L^1^** (0.6 mg/kg) and PBS. (e) The tumor weight of mice injected with compound **L^1^** (0.6 mg/kg) and PBS control after 25 days. (f) H&E staining of the brain, heart, liver, spleen, lung, and kidney tissues collected from mice on the 25th day after an intravenous injection of compound **L^1^** (0.6 mg/kg) and PBS control. (g) CD31 immumohistochemical staining with mice on the 25th day after an intravenous injection of compound **L^1^** (0.6 mg/kg) and PBS control. Scale bar = 20 μm; error bars are based on standard errors of the mean (*n* = 5).

As shown in Figure 7(a–f), it is obvious that the volume and weight of tumor mice were decreased after injected with compound **L^1^** (0.6 mg/kg) as compared with PBS control. The average volume of tumor (HepG2) was 0.739 cm^3^ when the PBS control was 1.876 cm^3^. In addition, the weight of tumor was 0.84 g compared with PBS control was 2.16 g. The relative tumor proliferation rate (T/C) was 38.6% and the tumor inhibition rate was up to 61.2%. Moreover, no obvious toxicity was also observed in the heart, liver, spleen, lung, kidney, and brain tissues of the mice injected with compound **L^1^** in Figure 7(f), which exhibited no significant changes in morphology of these organs. In Figure 7(g), CD31 immumohistochemical staining with mice was taken on the 25th day after an intravenous injection of compound **L^1^** (0.6 mg/kg) showed tumor angiogenesis rate was decreased compared with PBS control, which demonstrated compound **L^1^** could suppress tumor growth. In general, these findings suggested that **L^1^** had high anti-HepG2 activity both *in vitro* and *in vivo*, and **L^1^** had great promising future as nontoxic side effects and effective antiproliferation drug.

## 3. Conclusions

In conclusion, our formulation has shown thiophene Schiff-bases (**L^1^**−**L^3^**), thiophene amides (**L^4^**−**L^6^**) and pyrazine amides (**L^7^**−**L^10^**) with high antiproliferative activity, relatively low toxicity and DNA binding modes. The toxicity of **L^1^**−**L^10^**(IC_50_ = 5.92− >100 μM) was all lower than **L^0^** (1.27 μM) and DOX (4.40 μM). Compound **L^1^** had higher anti-HepG2 **(**0.66 μM), anti-MCF-7 (5.33 μM) and anti-A549 (2.11 μM) activity. Compound **L^3^** had higher anti-HepG2 (1.63 μM) and anti-MCF-7 (2.65 μM) activity. Additionally, **L^1^** and **L^3^** were verified with high efficiency apoptosis induction in HepG2 cells and intercalated modes of binding with DNA. **L^1^** had no significant toxicity but high anti-HepG2 activity both *in vitro* and *in vivo*; it may be an effective antiproliferation drug with nontoxic side effects.

These findings can provide a convenient procedure of the rational design in new and potential cellular targeting compounds, which may help to explore the future selective anticancer drugs directly toward cellular targets. However, the possible cellular and structural mechanisms are still unclear and its investigation is currently ongoing.

## 4. Experimental section

### 4.1. General experimental procedures

All reagents and solvents were analytical reagent (AR) grade and used as received unless otherwise indicated. The IR spectra were recorded on a Bruker Vectex 80 FT − IR spectrometer with KBr discs in the 4000–500 cm^−1^ range. The ^1 ^H NMR spectra were measured on a Bruker Avance III 600 MHz NMR spectrometer (Billerica, MA, USA). ^1 ^H and ^13 ^C {^1 ^H} NMR spectra were recorded in CDCl_3_ as solvent unless otherwise stated. Chemical shifts (*δ*) were given as parts per million (ppm) relative to the NMR solvent signals (CDCl_3_ 7.26 and 77.00 ppm for ^1 ^H and ^13 ^C{^1^H} NMR, respectively). *J* values were given in Hz. HRMS were measured to determine purity of all tested compounds by LTQ Orbitrap XL mass spectrometer (Thermo Electron, USA). Reactions were monitored by TLC using silica gel 60 F–254 in 0.25 mm thick plates. Compounds on TLC plates were detected under UV light at 254 nm. Purifications were performed by flash chromatography on silica gel (300–400 mesh). The DNA binding modes were investigated by Lambda 950 (Perkin Elmer, USA) and LS55 Fluorescence Spectrophotometer (Perkin Elmer, USA).

### 4.2. Synthesis of compound L^1^−L^10^

Thiophene Schiff-bases (**L^1^**−**L^3^**), thiophene amides (**L^4^**−**L^6^**), and pyrazine amides (**L^7^**−**L^10^**) were synthesized by the following methods.

#### 4.2.1. Synthesis of 2-thiophene-dehydroabietylamine-Schiff-base (L^1^)

**L^0^** (1.43 g, 5.0 mmol), 2-thiopehne-formaldehyde (0.56 g 5.0 mmol) with acetic acid as the catalyst was dissolved in ethanol (50 mL) and refluxed for 24 h. When reaction mixture was cooled to the room temperature, lots of white needlelike crystal precipitation appeared. Then white needlelike crystals were obtained by recrystallization from ethanol solution. (1.58 g, 83%), mp: 83.6-85.4 °C; IR (neat) *ν*_max_/cm^−1^ 3423, 2929, 2861, 1649, 1598, 1450, 1375, 1036, 977, 747, 637; ^1 ^H-NMR (CDCl_3_, 600 MHz) *δ* 1.04 (3 H, s), 1.21-1.23 (9 H, t, *J* = 7.2 Hz), 1.30-1.50 (3 H, m), 1.60-1.66 (2 H, m), 1.71–1.79 (2 H, m), 1.88–1.90 (1 H, m), 2.24–2.26 (1 H, d, *J* = 12 Hz); 2.79–2.81 (2 H, m), 2.81–2.86 (1 H, m), 3.40 (2 H, m), 6.86 (1 H, s), 6.98 (1 H, d, *J* = 7.8 Hz), 7.02 (1 H, dd, *J* = 3.6 Hz), 7.17 (1 H, d, *J* = 8.4 Hz), 7.25 (1 H, d, *J* = 3.0 Hz), 7.31–7.32 (1 H, m), 8.30 (1 H, s); ^13 ^C{^1^H} (CDCl_3_, 151 MHz) *δ* 18.95, 19.45, 24.04, 24.05, 25.72, 30.58, 33.46, 36.72, 37.73, 38.27, 38.48, 46.11, 123.83, 124.48, 126.88, 127.26, 128.52, 129.70, 135.01, 143.02, 145.39, 147.51, 153.95; MS [M + H]^+^ *m/z* 380.2419 (calcd for C_25_H_33_NS 379.2334). Anal. calcd for C_25_H_33_NS: C, 79.10; H, 8.76; N, 3.69; S, 8.45. Found: C, 79.21; H, 8.62; N, 3.81; S, 8.36.

#### 4.2.2. Synthesis of 3-methyl-2-thiophene-dehydroabietylamine-Schiff-base (L^2^)

When the mixture was cooled to the room temperature, removed the solvent by reduced pressure distillation, and received the brown oil compound. Finally, the brown products were obtained by recrystallization from methanol solution and dried in vacuum. (1.34 g, 68%), mp: 38.2–39.1 °C; IR (neat) *ν*_max_/cm^−1^ 3428, 2929, 2868, 1629, 1447, 1378, 1207, 1050, 825, 716, 620; ^1 ^H–NMR (CDCl_3_, 600 MHz) *δ* 1.02 (3 H, s), 1.21–1.24 (9 H, t, *J* = 7.2 Hz), 1.38–1.52 (3 H, m), 1.60–1.66 (2 H, m), 1.69–1.80 (2 H, m), 1.91–1.95 (1 H, m), 2.25–2.27 (1 H, d, *J* = 11.4 Hz), 2.37(3 H, s), 2.80–2.87 (3 H, m), 3.41 (2 H, m), 6.83 (1 H, s), 6.98 (1 H, d, *J* = 7.2 Hz), 7.17 (1 H, dd, *J* = 8.4 Hz), 7.22 (1 H, d, *J* = 4.8 Hz), 7.24–7.25 (1 H, m), 8.36 (1 H, s); ^13 ^C{^1^H} ((CD_3_)_2_SO, 151 MHz) *δ* 15.79, 18.50, 18.69, 24.37, 24.45, 25.45, 29.46, 33.38, 35.02, 36.08, 37.40, 38.01, 40.41, 44.88, 50.04, 123.96, 124.36, 126.03, 126.81, 129.54, 134.89, 141.97, 142.87, 145.45, 147.28, 165.57; MS [M + H]^+^ *m/z* 394.5419 (calcd for C_26_H_35_NS 393.2490). Anal. calcd for C_26_H_35_NS: C, 79.33; H, 8.96; N, 3.56; S, 8.15. Found: C, 79.02; H, 8.69; N, 3.75; S, 8.54.

#### 4.2.3. Synthesis of 5-bromine-2-thiophene-dehydroabietylamine-Schiff-base (L^3^)

After the reaction mixture cooling to the room temperature, lots of pale yellow needlelike crystal precipitation appeared. Then faint yellow block-shaped single crystals were obtained by recrystallization from ethanol solution. (1.99 g, 87%), mp: 132.9–134.3 °C; IR (neat) *ν*_max_/cm^−1^ 3394, 2977, 2922, 1630, 1427, 1378, 1085, 1044, 879, 797, 620; ^1 ^H–NMR (CDCl_3_, 600 MHz) *δ* 1.04 (3 H, s), 1.21–1.23 (9 H, t, *J* = 7.2 Hz), 1.30–1.50 (3 H, m), 1.60–1.66 (2 H, m), 1.71–1.79 (2 H, m),1.88–1.90 (1 H, m), 2.24–2.26 (1 H, d, *J* = 12 Hz); 2.79–2.81 (2 H, m), 2.81–2.86 (1 H, m), 3.40 (2 H, dd, *J* = 12 Hz), 6.89 (1 H, s), 6.99–7.01 (3 H, m), 7.19–7.20 (1 H, d, *J* = 8.4 Hz), 8.19 (1 H, s); ^13 ^C{^1^H} (CDCl_3_, 151 MHz) *δ* 18.92, 18.95, 19.40, 24.00, 25.63, 30.54, 33.45, 36.74, 37.70, 38.24, 38.49, 46.15, 72.94, 116.61, 123.83, 124.43, 126.86, 129.52, 130.19, 134.89, 144.72, 145.39, 147.42, 153.02; MS [M + H]^+^ *m/z* 458.1526 (calcd for C_25_H_32_BrNS 457.1439). Anal. calcd for C_25_H_32_BrNS: C, 65.49; H, 7.03; N, 3.05; S, 6.99. Found: C, 65.34; H, 7.12; N, 3.11; S, 6.90.

#### 4.2.4. Synthesis of 2–thiophene-acyl-dehydroabietylamine (L^4^)

Equal 2-thiophene-carboxylic acid (0.64 g, 5.0 mmol) and HOBT (0.68 g, 5.0 mmol) were dissolved in ethyl acetate (40 mL), stirred for 0.5 h at 0 °C. Then DCC (1.03 g, 5.0 mmol) was slowly added, stirred for 2.5 h at 0 °C. The ethyl acetate solution (10 mL) of **L^0^** (1.43 g, 5.0 mmol) was added to the reaction system slowly, the mixture was stirred at room temperature for 8 h. Filter to remove DCU, filtrate was diluted to 200 mL and washed with 5% NaHCO_3_ (3*20 mL), 10% citric acid (2*20 mL) and saturated salt water. Ethyl acetate layer was dried with anhydrous Na_2_SO_4_ for 2 h, and solvents were evaporated to get milky white powders. Then colorless block-shaped single crystals were obtained by recrystallization from ethanol solution. (1.74 g, 88%), mp: 153.7–156.0 °C; IR (neat) *ν*_max_/cm^−1^ 3428, 2922, 2860, 1638, 1561, 1439, 1371, 1044, 976, 771, 606; ^1 ^H NMR (CDCl_3_, 600 MHz): *δ* 1.05 (3 H, s), 1.21–1.24 (9 H, s), 1.38–1.44 (2 H, m), 1.56–1.57 (1 H, m), 1.60–1.67 (2 H, m), 1.71–1.81 (2 H, m), 1.89–1.92 (1 H, m), 2.25–2.30 (1 H, m), 2.76–2.91 (3 H, m), 3.42–3.61 (2 H, dd, *J* = 12 Hz), 6.87 (1 H, s), 6.98 (1 H, dd, *J* = 7.2 Hz), 7.14 (1 H, d, *J* = 7.2 Hz), 7.18 (1 H, d, *J* = 8.4 Hz), 7.29–7.30 (1 H, m), 7.69–7.72 (1 H, m), 8.01–8.05 (1 H, m), 8.34 (1 H, s), 8.61(1 H, d, *J* = 3.6 Hz); ^13 ^C{^1^H} NMR (CDCl_3_, 151 MHz): *δ* 18.76, 18.90, 19.64, 23.96, 25.64, 30.48, 33.40, 36.60, 37.66, 38.20, 38.42, 45.63, 58.45, 72.91, 121.16, 123.80, 124.41, 124.61, 126.87, 135.00, 136.55, 145.39, 147.47, 149.18, 154.68, 161.83; MS [M + H]^+^ *m/z* 375.2800 (calcd for C_26_H_34_N_2_ 374.2722). Anal. calcd for C_26_H_34_N_2_: C, 83.37; H, 9.15; N, 7.48. Found: C, 83.43; H, 9.28; N, 7.29.

#### 4.2.5. Synthesis of 5-methyl-2-thiophene-acyl-dehydroabietylamine (L^5^)

The condensation reaction used HOBT and DCC like the method of **L^4^**. The solution was dried with anhydrous Na_2_SO_4_ and evaporated to get milky white powders. (1.72 g, 84%), mp: 169.4–171.7 °C; IR (neat) *ν*_max_/cm^−1^ 3413, 2922, 2854, 1629, 1553, 1453, 1290, 1050, 1046, 811, 743, 606; ^1 ^H–NMR ((CD_3_)_2_SO, 600 MHz) *δ* 0.90 (3 H, s), 1.13–1.15 (9 H, t, *J* = 7.2 Hz), 1.32–1.36(2H, m), 1.43–1.45 (1 H, m), 1.56–1.61(2H, m), 1.69–1.71 (2 H, m), 1.96–1.99 (1 H, m), 2.24 (1 H, d, *J* = 12.6 Hz), 2.42–2.44 (3 H, s), 2.73–2.78 (3 H, m), 2.90–3.40 (2 H, dd, *J* = 7.2 Hz), 6.77 (1 H, s), 6.82 (1 H, d, *J* = 1.8 Hz), 6.92 (1 H, dd, *J* = 8.4 Hz), 7.12 (1 H, d, *J* = 8.4 Hz), 7.60 (1 H, d, *J* = 3.6 Hz), 8.09 (1 H, s, –NH); ^13 ^C{^1^H} NMR ((CD_3_)_2_SO, 151 MHz) *δ* 15.61, 18.74, 19.13, 19.30, 24.35, 24.40, 25.82, 30.37, 33.35, 36.35, 37.48, 38.32, 45.04, 49.72, 123.93, 124.46, 126.64, 126.88, 128.64, 135.00, 138.10, 144.73, 145.28, 147.44, 161.88; MS [M + H]^+^ *m/z* 410.2527, MS [M + Na]^+^ *m/z* 432.2346 (calcd for C_26_H_35_NOS 409.2439). Anal. calcd for C_26_H_35_NOS: C, 76.24; H, 8.61; N, 3.42; S, 7.83. Found: C, 76.19; H, 8.72; N, 3.36; S, 7.74.

#### 4.2.6. Synthesis of 5-bromine-2-thiophene-acyl-dehydroabietylamine (L^6^)

HOBT and DCC were used in the condensation reaction (same as **L^4^**). Ethyl acetate was dried with and evaporated to get milky white powders. (1.92 g, 81%), mp: 170.7–173.2 °C; IR (neat) *ν*_max_/cm^−1^ 3414, 2922, 2849, 1629, 1550, 1413, 1282, 1077, 805, 736, 620; ^1 ^H–NMR ((CD_3_)_2_SO, 600 MHz) *δ* 0.90 (3 H, s), 1.13–1.15 (9 H, t, *J* = 6.6 Hz), 1.32–1.35 (2 H, m), 1.42–1.46 (1 H, m), 1.57–1.61(2H, m), 1.62–1.73 (2 H, m), 1.95–1.99 (1 H, m), 2.24 (1 H, d, *J* = 12.6 Hz), 2.72–2.79 (3 H, m), 2.92–3.41 (2 H, m), 6.82 (1 H, s), 6.92 (1 H, dd, *J* = 7.8 Hz), 7.12 (1 H, d, *J* = 7.8 Hz), 7.22 (1 H, d, *J* = 3.6 Hz), 7.68 (1 H, d, *J* = 4.2 Hz), 8.33 (1 H, s, –NH); ^13 ^C{^1^H} NMR ((CD_3_)_2_SO, 151 MHz) *δ* 18.71, 19.12, 19.26, 24.35, 24.40, 25.81, 30.34, 33.34, 36.35, 37.48, 38.34, 40.42, 45.78, 45.08, 49.87, 117.02, 123.94, 124.46, 126.89, 129.26, 131.81, 134.95, 142.47, 145.29, 147.40, 160.88; MS [M + H]^+^ *m/z* 475.3262 (calcd for C_25_H_32_BrNOS 474.3138). Anal. calcd for C_25_H_32_BrNOS: C, 63.28; H, 6.80; N, 2.95; S, 6.76. Found: C, 63.19; H, 6.87; N, 2.83; S, 6.82.

#### 4.2.7. Synthesis of 2-pyrazine-acyl-dehydroabietylamine (L^7^)

After the condensation reaction of 2-pyrazine-carboxylic acid (0.62 g, 5.0 mmol) and dehydroabietylamine (1.43 g, 5.0 mmol) used HOBT and DCC, which was same as **L^4^**, solvents were evaporated to get yellow powders. (1.53 g, 78%), mp: 159.8–162.3 °C; IR (neat) *ν*_max_/cm^−1^ 3401, 2929, 2854, 1653, 1524, 1392, 1153, 1016, 873, 819, 620; ^1 ^H–NMR (CDCl_3_, 600 MHz) *δ* 1.02 (3 H, s), 1.21–1.23 (9 H, t, *J* = 7.2 Hz), 1.38–1.41 (1 H, m), 1.51–1.53 (2 H, m), 1.66–1.71(1H, m), 1.77–1.79 (2 H, m), 1.91–2.04 (2 H, m), 2.29 (1 H, d, *J* = 12.6 Hz), 2.80–2.89 (2 H, m), 2.90–2.95(1H, m), 3.26–3.54 (2 H, dd, *J* = 7.2 Hz), 6.89 (1 H, s), 6.98 (1 H, dd, *J* = 8.4 Hz), 7.16 (1 H, d, *J* = 7.8 Hz), 7.91 (1 H, s, –NH), 8.49 (1 H, d, *J* = 1.2 Hz), 8.72 (1 H, d, *J* = 2.4 Hz), 9.39 (1 H, s); ^13 ^C{^1^H} NMR ((CD_3_)_2_SO, 151 MHz) *δ* 18.43, 18.68, 24.38, 24.44, 25.43, 29.41, 33.36, 35.02, 36.04, 37.40, 37.99, 44.89, 50.15, 123.99, 124.37, 126.79, 134.85, 143.90, 144.75, 145.50, 145.57, 147.26, 151.88, 167.45; MS [M + H]^+^ *m/z* 392.2721, [M + Na]^+^ *m/z* 414.2539 (calcd for C_25_H_33_N_3_O 391.2624). Anal. calcd for C_25_H_33_N_3_O: C, 76.69; H, 8.49; N, 10.73. Found: C, 76.57; H, 8.56; N, 10.64.

#### 4.2.8. Synthesis of 5-methoxyl-2-pyrazine-acyl-dehydroabietylamine (L^8^)

A mixture of Dehydroabietylamine (**L^0^**) (10.0 mmol), 5-chloro-pyrazine-2-carboxylic acid methyl ester (10.0 mmol) was dissolved in EtOH (150 mL) and refluxed for 24 h. Then the mixture was cooled to the room temperature, removed the solvent by reduced pressure distillation, and received the orange powders. Finally, the orange powders were obtained by recrystallization from ethanol solution. (1.37 g, 65%), mp: 117.6–119.8 °C; IR (neat) *ν*_max_/cm^−1^ 3422, 2929, 2868, 1714, 1593, 1433, 1276, 1125, 1016, 825, 620; ^1 ^H–NMR ((CD_3_)_2_SO, 600 MHz) *δ* 0.92 (3 H, s), 1.11–1.21 (9 H, t, *J* = 7.2 Hz), 1.22–1.26 (2 H, m), 1.38–1.49 (2 H, m), 1.52–1.64 (2 H, m), 1.64–1.72 (1 H, m), 1.88–1.92 (1 H, m), 2.23 (1 H, d, *J* = 12.0 Hz), 2.63–2.79 (3 H, m), 3.16–3.53 (2 H, m), 3.79 (3 H, s), 6.79 (1 H, s), 6.92 (1 H, dd, *J* = 7.8 Hz), 7.11 (1 H, d, *J* = 7.8 Hz), 7.80 (1 H, s, –NH), 8.08 (1 H, s), 8.59 (1 H, s); ^13 ^C{^1^H} NMR ((CD_3_)_2_SO, 151 MHz) *δ* 14.49, 18.73, 19.00, 19.25, 21.13, 24.31, 25.58, 29.97, 33.35, 36.02, 37.44, 44.35, 50.62, 51.86, 60.18, 123.92, 124.40, 126.82, 129.62, 134.04, 134.78, 145.32, 147.40, 157.09, 165.16; MS [M + H]^+^ *m/z* 422.2804, [M + Na]^+^ *m/z* 444.2620 (calcd for C_26_H_35_N_3_O_2_ 421.2729). Anal. calcd for C_26_H_35_N_3_O_2_: C, 74.07; H, 8.37; N, 9.97. Found: C, 74.01; H, 8.46; N, 9.89.

#### 4.2.9. Synthesis of 5–methyl-2-pyrazine-acyl-dehydroabietylamine (L^9^)

**L^9^** was synthesized according to the method used for **L^4^**. Obtained **L^9^**is pale yellow powders. (1.66 g, 82%), mp: 130.7–133.4 °C; IR (neat) *ν*_max_/cm^−1^ 3387, 2922, 2854, 1664, 1534, 1439, 1378, 1290, 1167, 1030, 819, 634; ^1 ^H–NMR (CDCl_3_, 600 MHz) *δ* 1.02 (3 H, s), 1.21–1.23 (9 H, t, *J* = 6.6 Hz), 1.38–1.42 (2 H, m), 1.51–1.53 (2 H, m), 1.67–1.73 (1 H, m), 1.70–1.81 (2 H, m),1.97–2.01 (1 H, m), 2.28 (1 H, d, *J* = 12.0 Hz), 2.63 (3 H, s), 2.81–2.94 (3 H, m), 3.25–3.49 (2 H, dd, *J* = 7.2 Hz), 6.88 (1 H, s), 6.98 (1 H, dd, *J* = 1.8 Hz), 7.16 (1 H, d, *J* = 7.2 Hz), 7.85(1 H, s, –NH), 8.34(1 H, d, *J* = 3.0 Hz), 9.25 (1 H, d, *J* = 1.8 Hz); ^13 ^C{^1^H} NMR (CDCl_3_, 151 MHz) *δ* 18.92, 19.12, 21.77, 23.94, 24.92, 25.47, 30.43, 33.91, 36.31, 37.58, 37.83, 38.28, 45.42, 49.75, 123.87, 124.24, 126.95, 134.84, 141.80, 142.22, 143.39, 145.61, 147.07, 156.91, 163.28; MS [M + H]^+^ *m/z* 406.2879, [M + Na]^+^ *m/z* 428.2698 (calcd for C_26_H_35_N_3_O 405.2780). Anal. calcd for C_26_H_35_N_3_O: C, 77.00; H, 8.70; N, 10.36. Found: C, 76.92; H, 8.62; N, 10.44.

#### 4.2.10. Synthesis of 5-chloro-2-pyrazine-acyl-dehydroabietylamine (L^10^)

The condensation reaction yielded pale yellow powders, which was synthesized according to the method used for **L^4^**. (1.59 g, 75%), mp: 152.4–154.5 °C; IR (neat) *ν*_max_/cm^−1^ 3387, 2929, 2860, 1664, 1529, 1447, 1282, 1167, 1125, 1022, 825, 620; ^1 ^H–NMR ((CD_3_)_2_SO, 600 MHz) *δ* 0.90 (3 H, s), 1.12–1.18 (9 H, t, *J* = 7.2 Hz), 1.32–1.42 (3 H, m), 1.57–1.76 (4 H, m), 2.01–2.04 (1 H, m), 2.24 (1 H, d, *J* = 13.8 Hz), 2.73–2.81 (3 H, m), 3.09–3.44 (2 H, dd, *J* = 6.6 Hz), 6.83 (1 H, s), 6.92 (1 H, dd, *J* = 7.8 Hz), 7.11 (1 H, d, *J* = 7.8 Hz), 7.12 (1 H, d, *J* = 8.4 Hz), 7.60 (1 H, d, *J* = 3.6 Hz), 8.60 (1 H, s, –NH), 8.83 (1 H, s), 9.00 (1 H, s); ^13 ^C{^1^H} NMR ((CD_3_)_2_SO, 151 MHz) *δ* 18.66, 19.08, 19.20, 24.30, 25.66, 30.18, 33.27, 36.31, 37.53, 38.36, 38.47, 40.62, 45.39, 49.76, 123.94, 124.36, 126.86, 134.91, 143.47, 143.83, 144.14, 145.35, 147.44, 151.13, 162.78; MS [M + Na]^+^ *m/z* 448.2146 (calcd for C_25_H_32_ClN_3_O 425.2234). Anal. calcd for C_25_H_32_ClN_3_O: C, 70.49; H, 7.57; N, 9.86. Found: C, 70.37; H, 7.48; N, 9.94.

### 4.3. Biological assays

#### 4.3.1. Cell culture, antiproliferative activities and cytotoxicity assay

Reagents and compounds Dulbecco’s Modified Eagle’s Medium (DMEM) and 3-(4,5-dimethylthiazol-2-yl)-2,5-diphenyltetrazolium bromide (MTT), fetal bovine serum (FBS) and penicillin/streptomycin were all commercially purchased.

Hela, HepG2, MCF-7, A549, and HUVEC cells were used in the antiproliferative activity assay. Cancer cells were seeded in 96-well plates with a density of 10^4^ cells per well, after 12 hours of incubation at 5% CO_2_ and 37 °C, the culture media was removed and cells were incubated with **L^1^−L^10^** dissolved in DMEM at different concentrations (each concentration repeated 3 times) for 36 h at 5% CO_2_ and 37 °C. Subsequently, removed the culture media and the new culture medium containing MTT (1 mg/mL) was added, followed by incubating for 4 h to allow the formation of formazan dye (Xia et al., 2015; Zhao et al., 2018). After removing the medium, 200 μL DMSO was added to each well to dissolve the formazan crystals. Absorbance was measured at 595 nm in a microplate photometer. Cell viability values were determined (at least three times) according to the following formulae: cell viability (%) = the absorbance of experimental group/the absorbance of blank control group ×100%.

#### 4.3.2. Induction of apoptosis by flow-cytometric analysis

Induction apoptosis assay was operated by Becton Dickinson Ultra-high speed separation flow-cytometry instrument and Annexin V-FITC/PI was purchased from Nanjing Keygen Biotech Co. Ltd.

We further investigated whether **L^1^** and **L^3^** could induce apoptosis; DMSO was used as negative control. HepG2 cells (1 × 10^6^) were cultured in 35 mm dishes and incubated at 37 °C for 24 h. After incubation with DMSO at 5 *μ*g/mL, **L^1^** at 1, 2, 5 *μ*g/mL, and **L^3^** at 0.1, 1, 5 *μ*g/mL for 24 h (each concentration repeated 3 times, the incubation time is optimum), the treated cells were washed, trypsinized (non-EDTA), and centrifuged (2000 rpm/min). Then the cells were collected and resuspended in 500 μL of buffer solutions loaded with Annexin V-FITC apoptosis detection reagent (with 5 μL Annexin V-FITC and 5 μL PI). The Annexin V-FITC-stained cells were incubated for 5–15 min in the dark, and approximate 10^4^ cells were collected for flow-cytometry analysis with a single 488 nm argon laser.

#### 4.3.3. In vivo experiment

*In vivo* experiment was taken by Nanjing Keygen Biotech Co. Ltd. For developing the tumor model, 1 × 10^6^ HepG2 cells were subcutaneously injected into the right armpit of every Balb/C nude mouse. And then two groups of HepG2-tumor-bearing mice with five mice per group were randomly chosen in our experiment: (1) PBS (as a control) and (2) compound **L^1^**. After the size of tumors reached 80 mm^3^, all agents including PBS, compound **L^1^**solutions were administrated via an intravenous injection (dose = 0.6 mg/kg), respectively. During the next 25 days, the tumor size of every mouse in our experiments was measured by a vernier caliper every 3 days. Moreover, to accurately evaluate the growth inhibition of tumors, the mice were sacrificed after 25 days, and then their tumors were collected, photographed, and weighed. In addition, the sections of tumor, heart, kidney, liver, lung, and spleen tissues of different groups harvested on the 25th day were observed using H&E staining, and then examined by a pathologist. The tumor size was calculated as the volume = 0.5 × (tumor length) × (tumor width)^2^. The inhibition efficiency of tumor growth was calculated according to the following equation:

inhibition  efficiency(%)=(1−the  weight  of  experimental  group/the  weight  ofcontrol  group)×100%

CD31 immumohistochemical staining with mice was conducted on the 25th day after an intravenous injection of compound **L^1^** (0.6 mg/kg) and PBS control.

## Supplementary Material

Supplemental Material
