# Supplementary material for: Synthesis of novel, DNA binding heterocyclic dehydroabietylamine derivatives as potential antiproliferative and apoptosis-inducing agents
Source: Drug Deliv. 2020 Jan 27;27(1):216–27. doi: 10.1080/10717544.2020.1716879 (PMC7034089; doi:10.1080/10717544.2020.1716879)
Supplement: Supplemental Material [file IDRD_A_1716879_SM4759.docx]

**Supplementary Materials**

**Synthesis of novel, DNA binding heterocyclic dehydroabietylamine derivatives as potential antiproliferative and apoptosis-inducing agents**

Fengyi Zhao^a,b,c^, Xu Sun^c,d^, Wen Lu^c^, Li Xu ^a, c*^, Jiuzhou Shi^c^, Shilong Yang^e^, Mengyi Zhou^e^, Fan Su^e^, Feng Lin^e^, Fuliang Cao^a,b^

^a^Co-Innovation Center for Sustainable Forestry in Southern China, Nanjing Forestry University

^b^College of Forestry, Nanjing Forestry University, Nanjing 210037, PR China

^c^College of Science, Nanjing Forestry University, Nanjing 210037, PR China

^d^College of Information Science and Technology, Nanjing Forestry University, Nanjing 210037, PR China

^e^Advanced Analysis and Testing Center, Nanjing Forestry University, Nanjing 210037, PR China

*Email: [xuliqby@njfu.edu.cn](mailto:xuliqby@njfu.edu.cn)

**Contents**

1. X-ray Crystal structure determination.
2. **Fig. 1** a. Molecular structure of **L^3^** (Hydrogen atoms omitted for clarity); b. The 1D chain structure formed by intermolecular hydrogen bonds.
3. **Fig. 2** a. Molecular structure of **L^4^** (Hydrogen atoms omitted for clarity); b. The 1D chain structure formed by intermolecular hydrogen bonds.
4. **Fig. S1-S20** ^1^H, ^13^C NMR Spectra

**X-ray Crystal structure determination.**

Diffraction data were collected on a Bruker D8 VENTURE PHOTON 100 diffractometer using a graphite-monochromated MoKα radiation (0.71073Å) at 293 K in the *ω-2θ* scan mode. In all cases, an empirical absorption correction by SADABS was applied to the intensity data. The structures were solved by direct methods and refined on F^2^ by full-matrix least-squares methods using the SHELXTL crystallographic software package. All non-hydrogen atoms were refined anisotropically with hydrogen atoms included in calculated positions (riding model). Crystallographic data for compound **L^3^** and **L^4^** is given in Table S1. CCDC 1507479 contain the supplementary crystallographic data for compound **L^3^**. CCDC 1881775 contain the supplementary crystallographic data for compound **L^4^**. This data can be obtained free of charge from The Cambridge Crystallographic Data Centre via [www.ccdc.cam.ac.uk/data_request/cif](http://www.ccdc.cam.ac.uk/data_request/cif).

**Table S1**

Crystallographic Data for **L^3^**.

| **L^3^** | | **L^4^** |
| --- | --- | --- |
| Empirical formula | C_25_H_32_BrNS | C_25_H_33_NOS |
| Formula weight | 458.48 | 395.58 |
| Crystal system | Monoclinic | Orthorhombic |
| Space group | *P*2_1_ | *P*2_1_ |
| *a* (Å) | 10.9317(5) | 7.0861(5) |
| *b* (Å) | 6.1152(3) | 9.7414(8) |
| *c* (Å) | 17.5239(9) | 31.842(2) |
| *α* (°) | 90 | 90 |
| *β* (°) | 99.971(3) | 90 |
| *γ* (°) | 90 | 90 |
| *V* (A^3^) | 1153.77(10) | 2198.0(3) |
| Z | 2 | 4 |
| *D*_calcd_ (g cm^-3^) | 1.320 | 1.195 |
| *μ* (mm^-1^) | 1.882 | 0.162 |
| Flack parameter | 0.009(8) | 0.08(5) |
| *F* (000) | 480 | 856 |
| *T* (K) | 293(2) | 273(2) |
| Crystal size(mm^3^) | 0.30×0.30× 0.10 | 0.30×0.20× 0.12 |
| ***θ*** Range(°) | 2.40-22.14 | 2.45-26.16 |
| Reflections collected | 9522 | 11355 |
| Independent reflections | 4695 | 4886 |
| Reflections observed [*I* > 2σ(*I*)] | 2645(*R*_int_=0.0401) | 3196(*R*_int_=0.0394) |
| Data/restraints/parameters | 4695/1/257 | 4886/1/257 |
| Goodness-of-fit on *F* ^2^ | 0.986 | 1.059 |
| Final *R* indices [*I* > 2σ(*I*)] | 0.0483/0.0827 | 0.0690/0.1374 |
| *R* indices (all data) | 0.1213/0.1004 | 0.1184/0.1578 |
| Largest difference peak and hole(eÅ^-3^) | 0.555, -0.221 | 0.408, -0.335 |

b

a


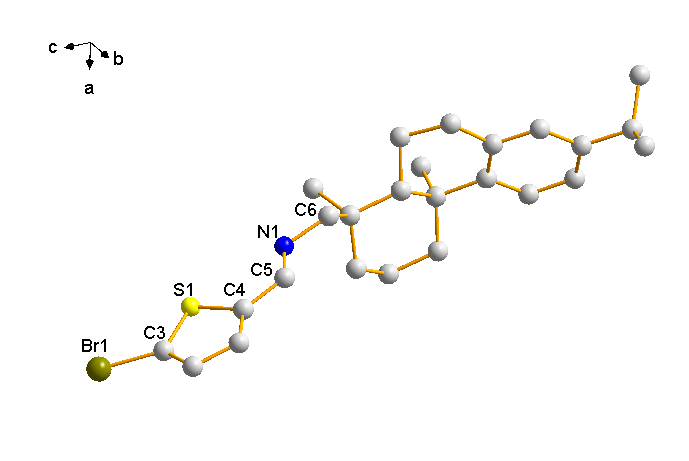

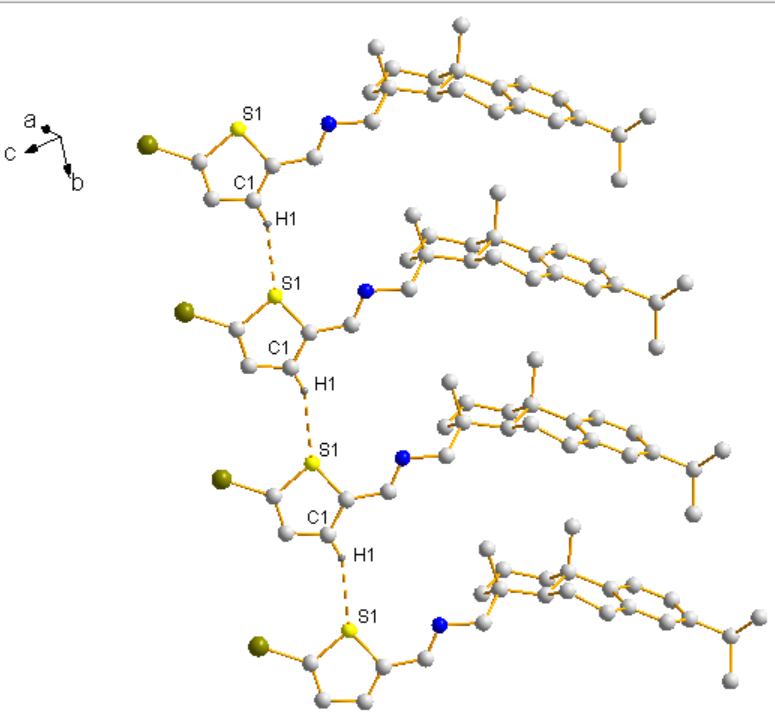


**Fig. 1** a. Molecular structure of **L^3^** (Hydrogen atoms omitted for clarity); b. The 1D chain structure formed by intermolecular hydrogen bonds.


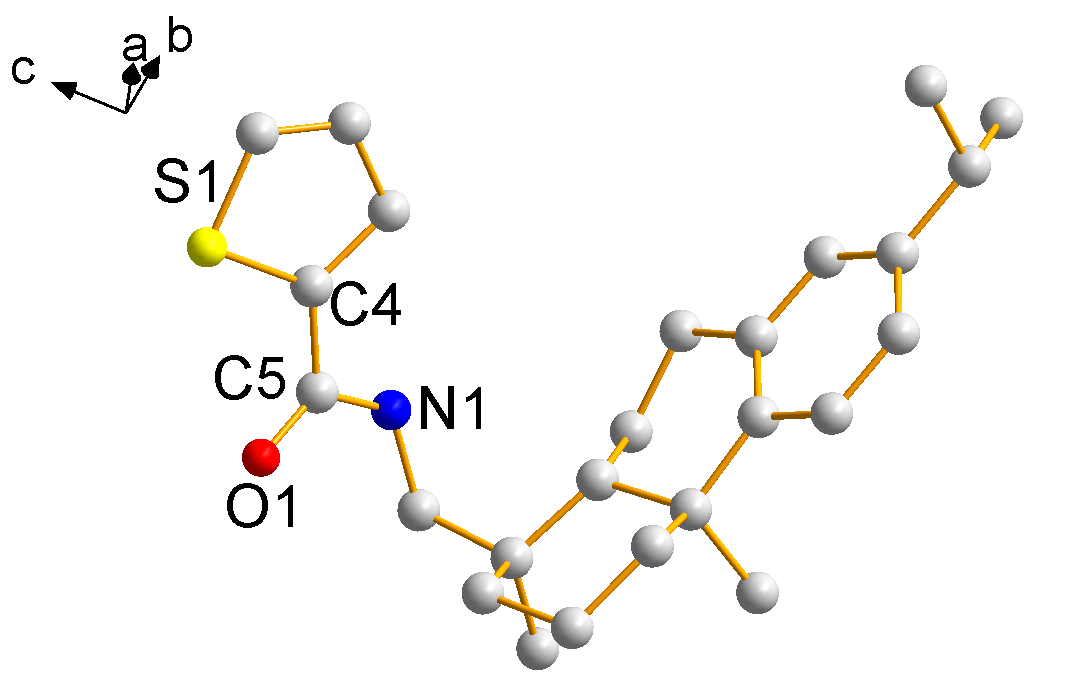

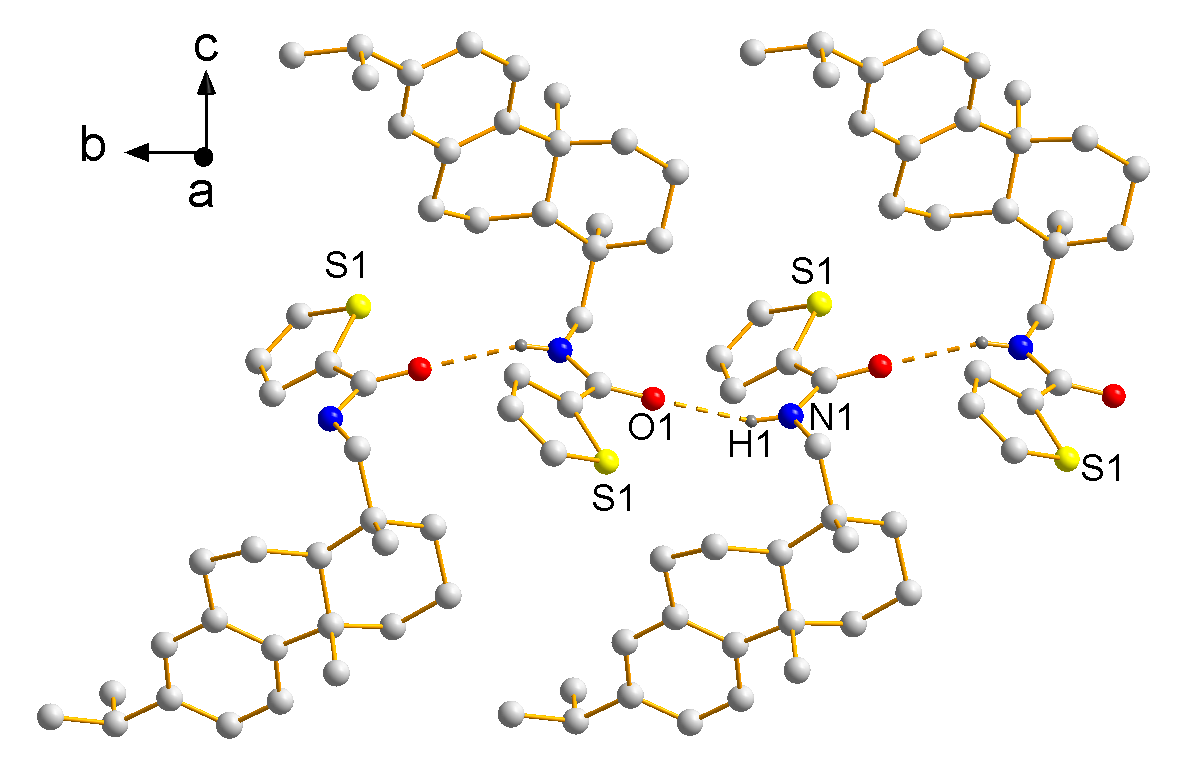


b

a

**Fig. 2** a. Molecular structure of **L^4^** (Hydrogen atoms omitted for clarity); b. The 1D chain structure formed by intermolecular hydrogen bonds.

^1^H NMR and ^13^C NMR spectra of compounds **L^1^**-**L^10^**.


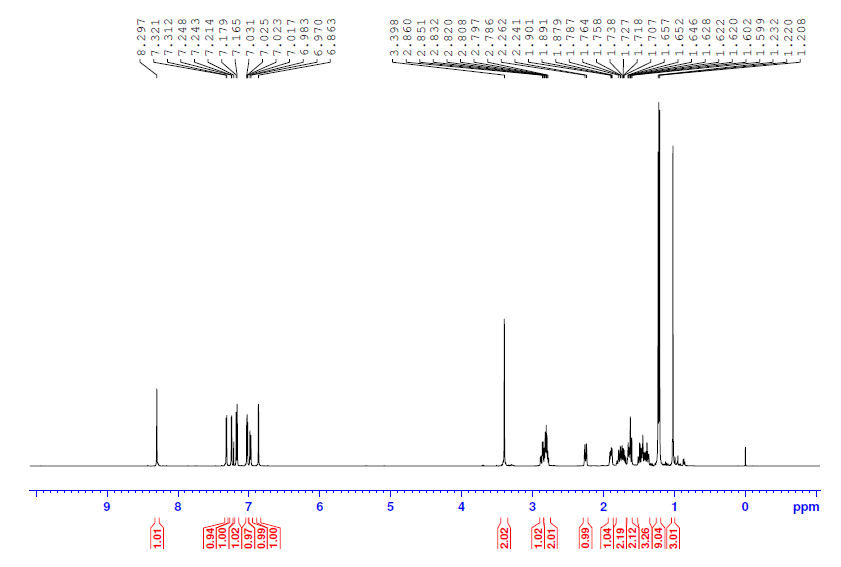


Fig.S1 ^1^H NMR (600 MHz, CDCl_3_) spectrum of compound **L^1^**

**^
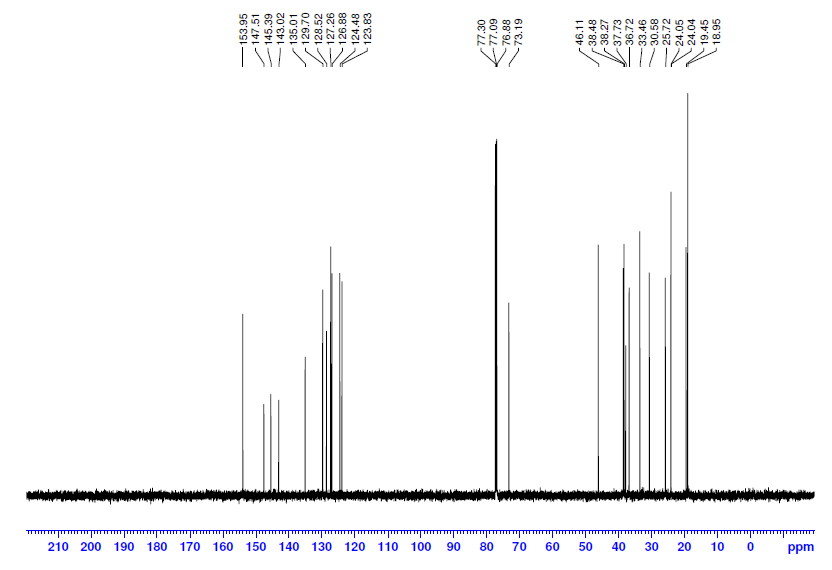
^**

Fig.S2 ^13^C NMR (151 MHz, CDCl_3_) spectrum of compound **L^1^**


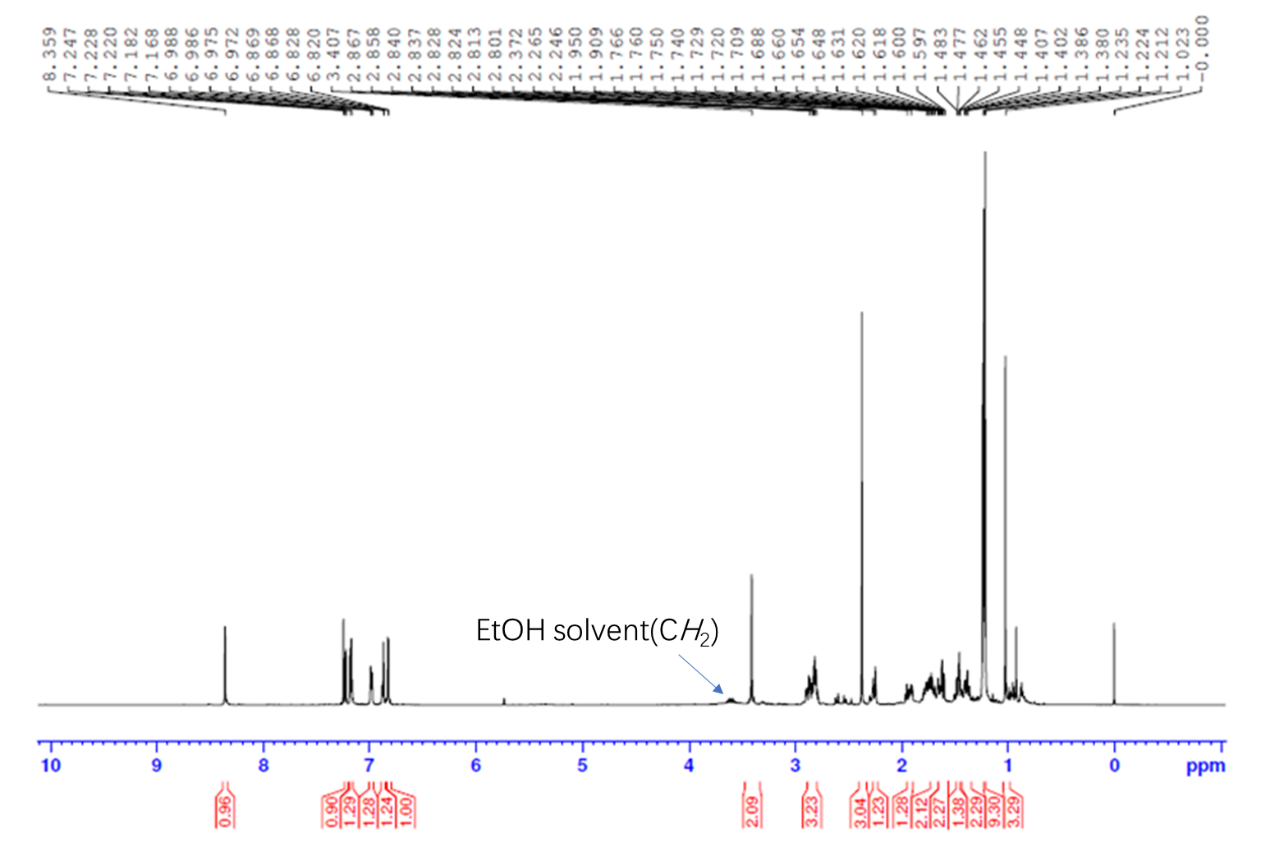


Fig.S3 ^1^H NMR (600 MHz, CDCl_3_) spectrum of compound **L^2^**


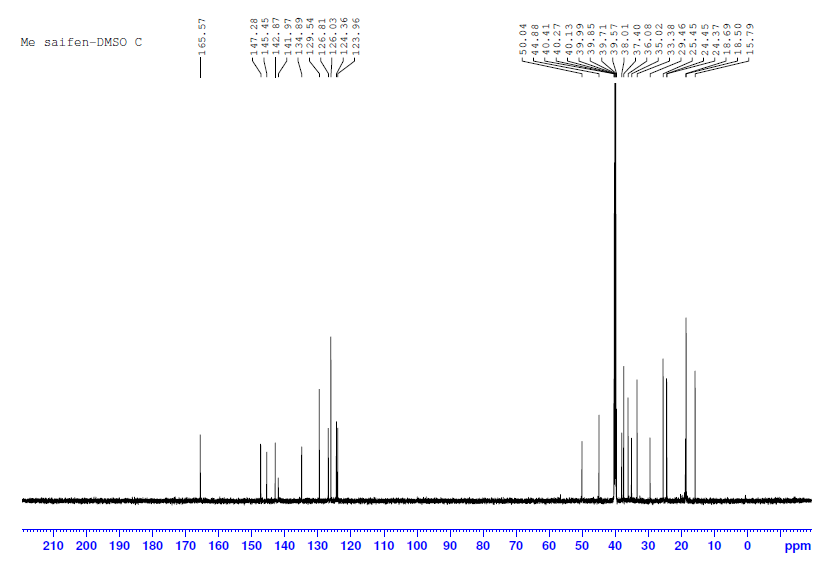


Fig.S4 ^13^C NMR (151 MHz, (CD_3_)_2_SO) spectrum of compound **L^2^**


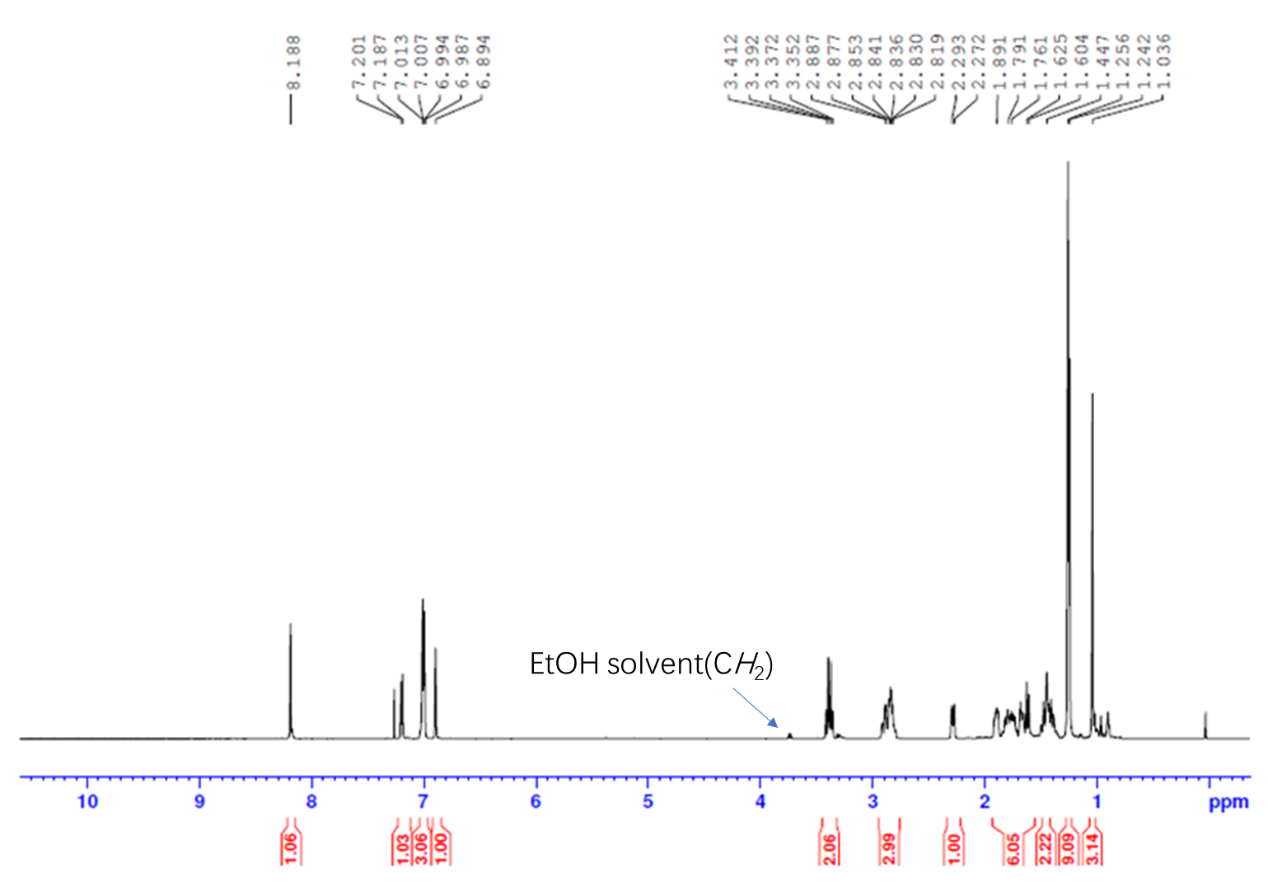


Fig.S5 ^1^H NMR (600 MHz, CDCl_3_) spectrum of compound **L^3^**

**^
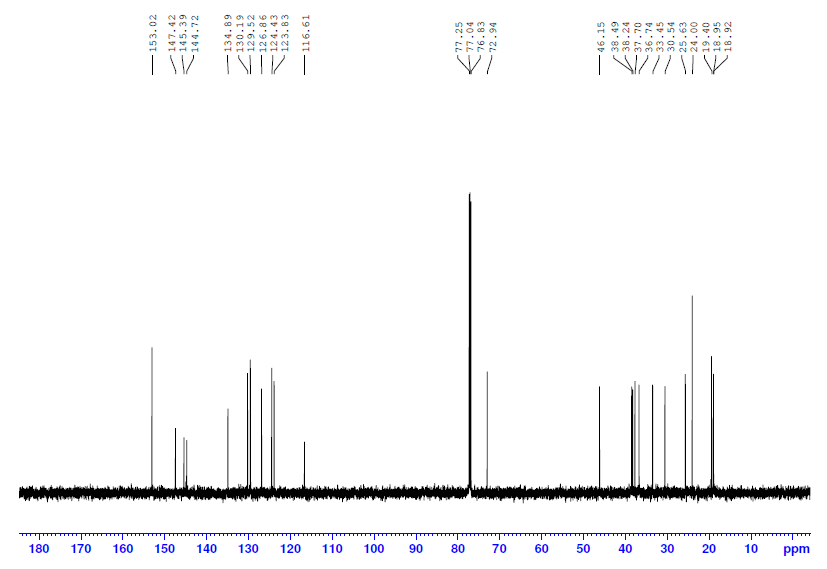
^**

Fig.S6 ^13^C NMR (151 MHz, CDCl_3_) spectrum of compound **L^3^**


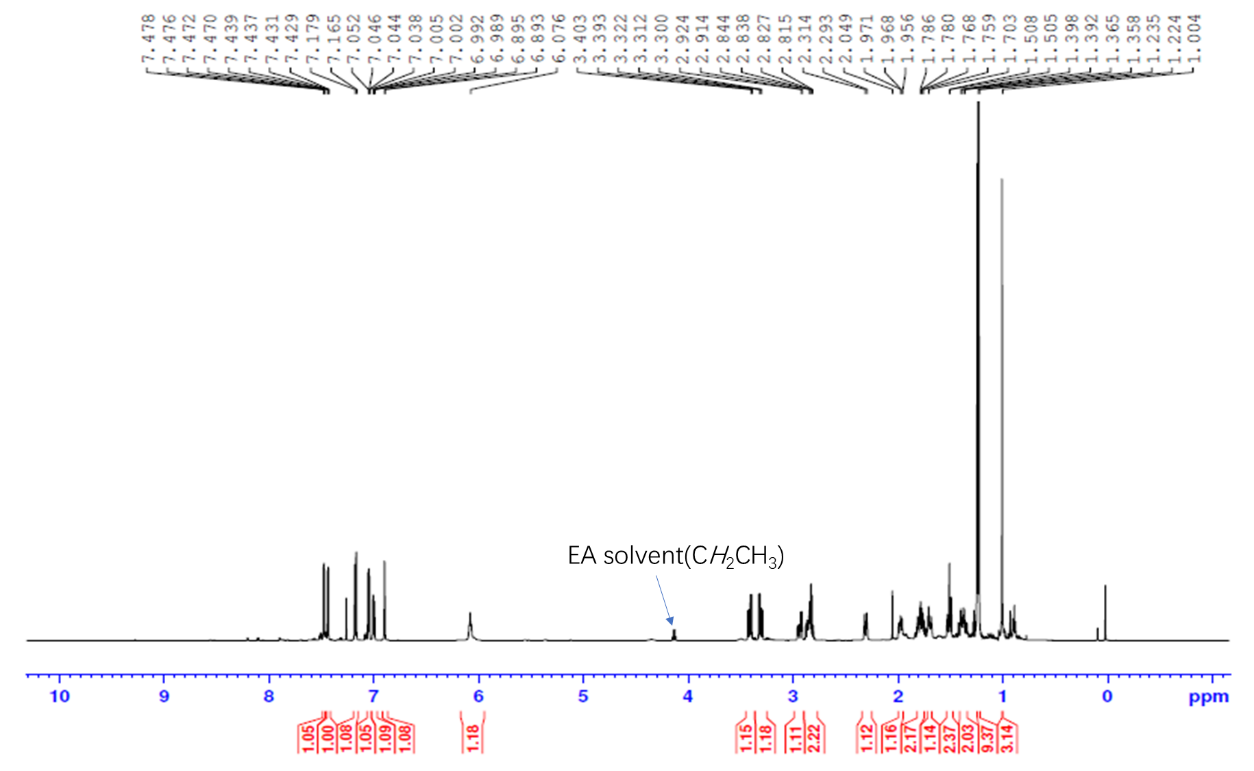


Fig.S7 ^1^H NMR (600 MHz, CDCl_3_) spectrum of compound **L^4^**


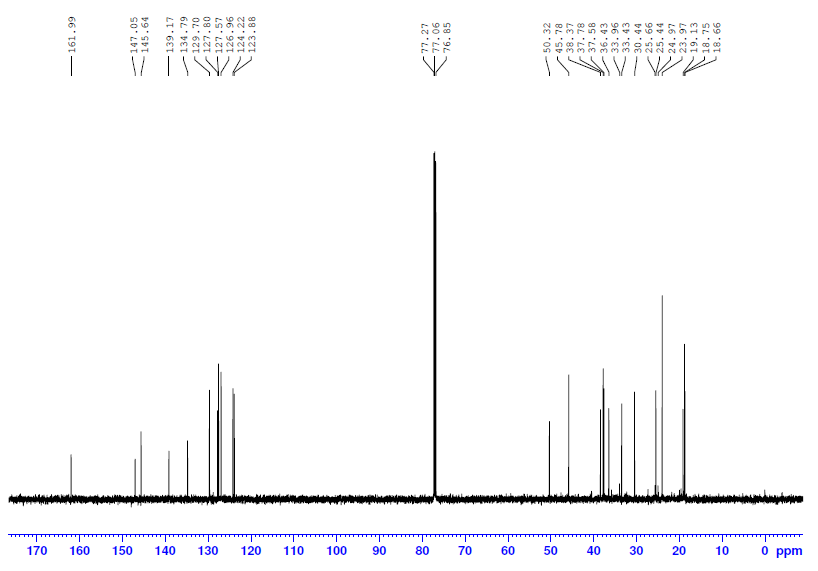


Fig.S8 ^13^C NMR (151 MHz, CDCl_3_) spectrum of compound **L^4^**


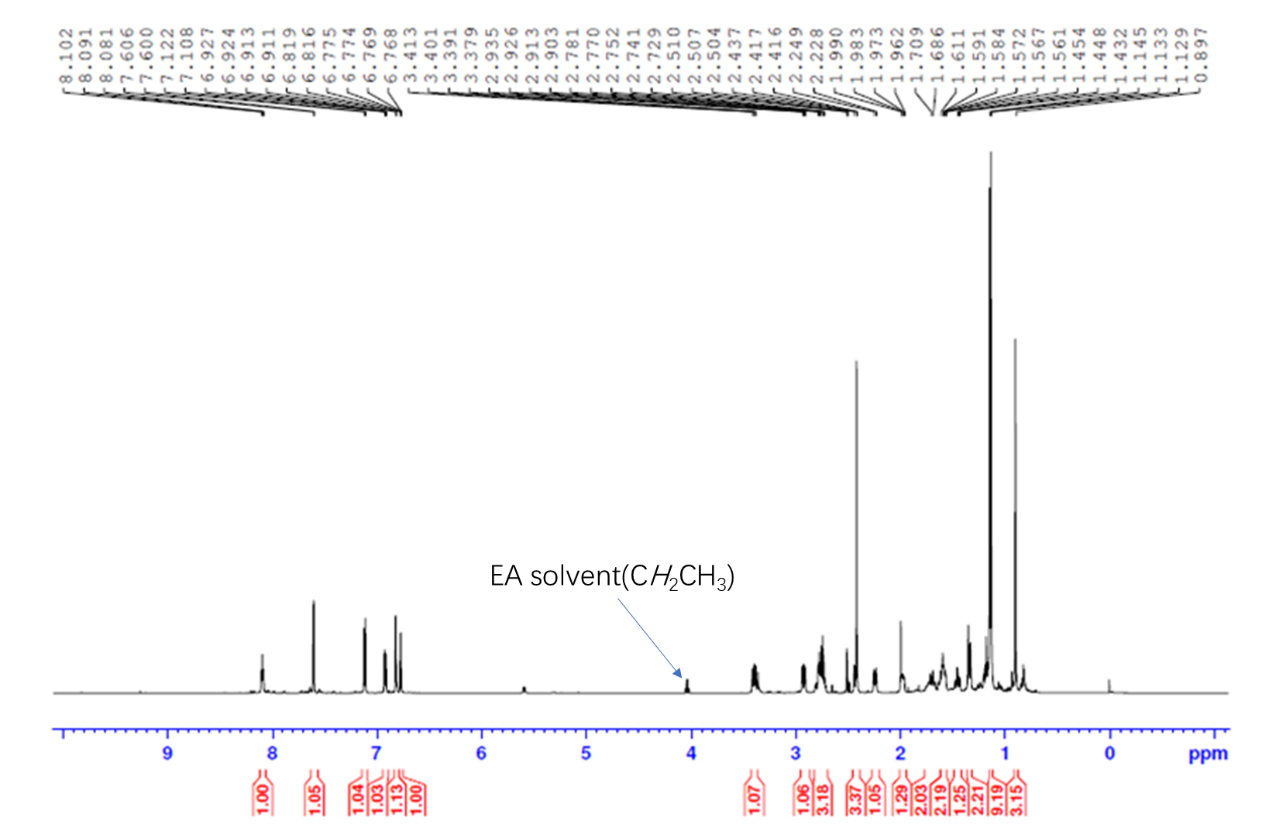


Fig.S9 ^1^H NMR (600 MHz, (CD_3_)_2_SO) spectrum of compound **L^5^**


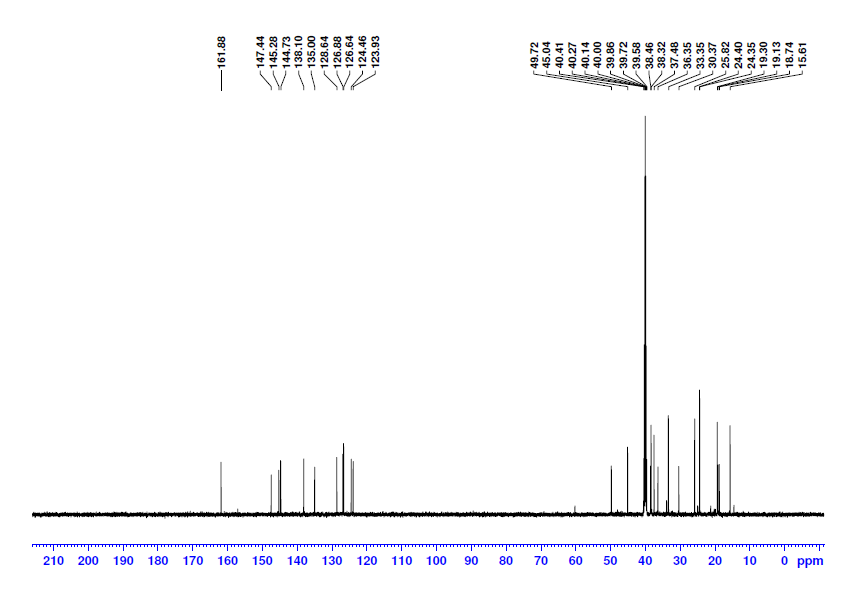


Fig.S10 ^13^C NMR (151 MHz, (CD_3_)_2_SO) spectrum of compound **L^5^**

**^
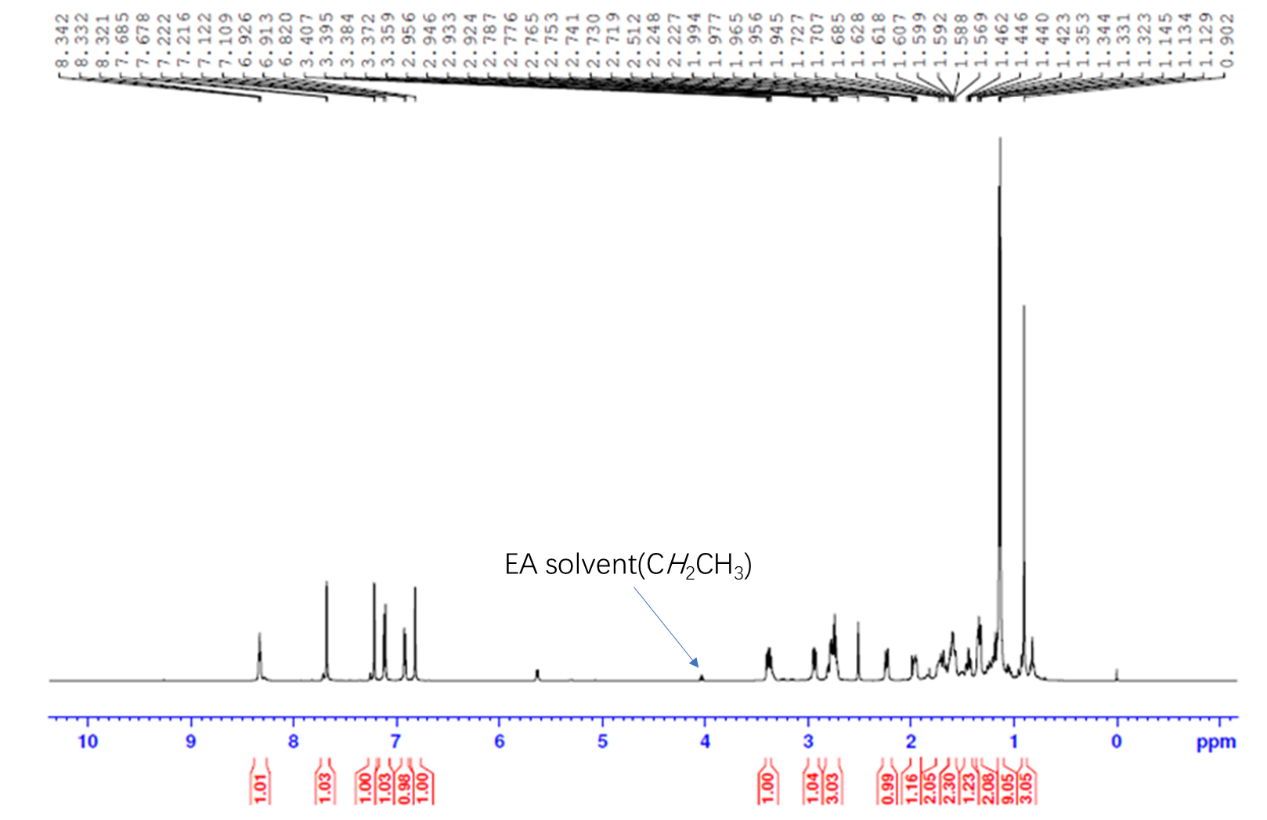
^**

Fig.S11 ^1^H NMR (600 MHz, (CD_3_)_2_SO) spectrum of compound **L^6^**

**^
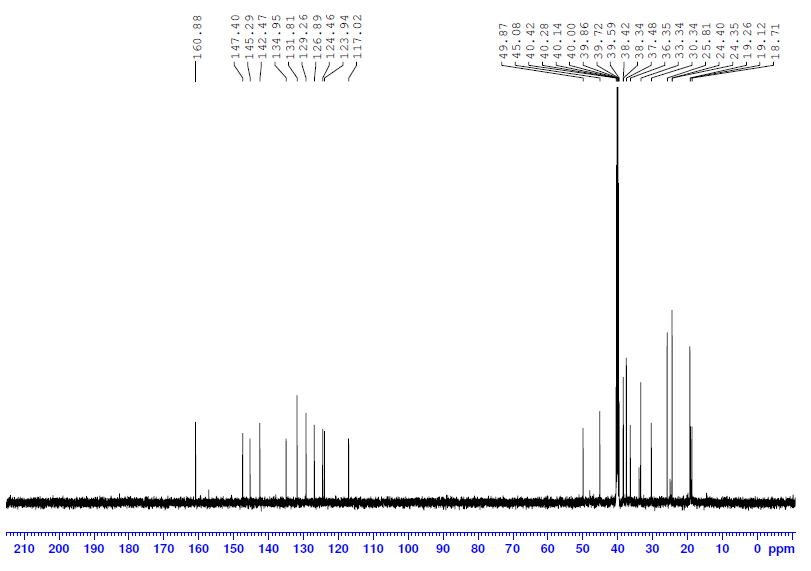
^**

Fig.S12 ^13^C NMR (151 MHz, (CD_3_)_2_SO) spectrum of compound **L^6^**

**^
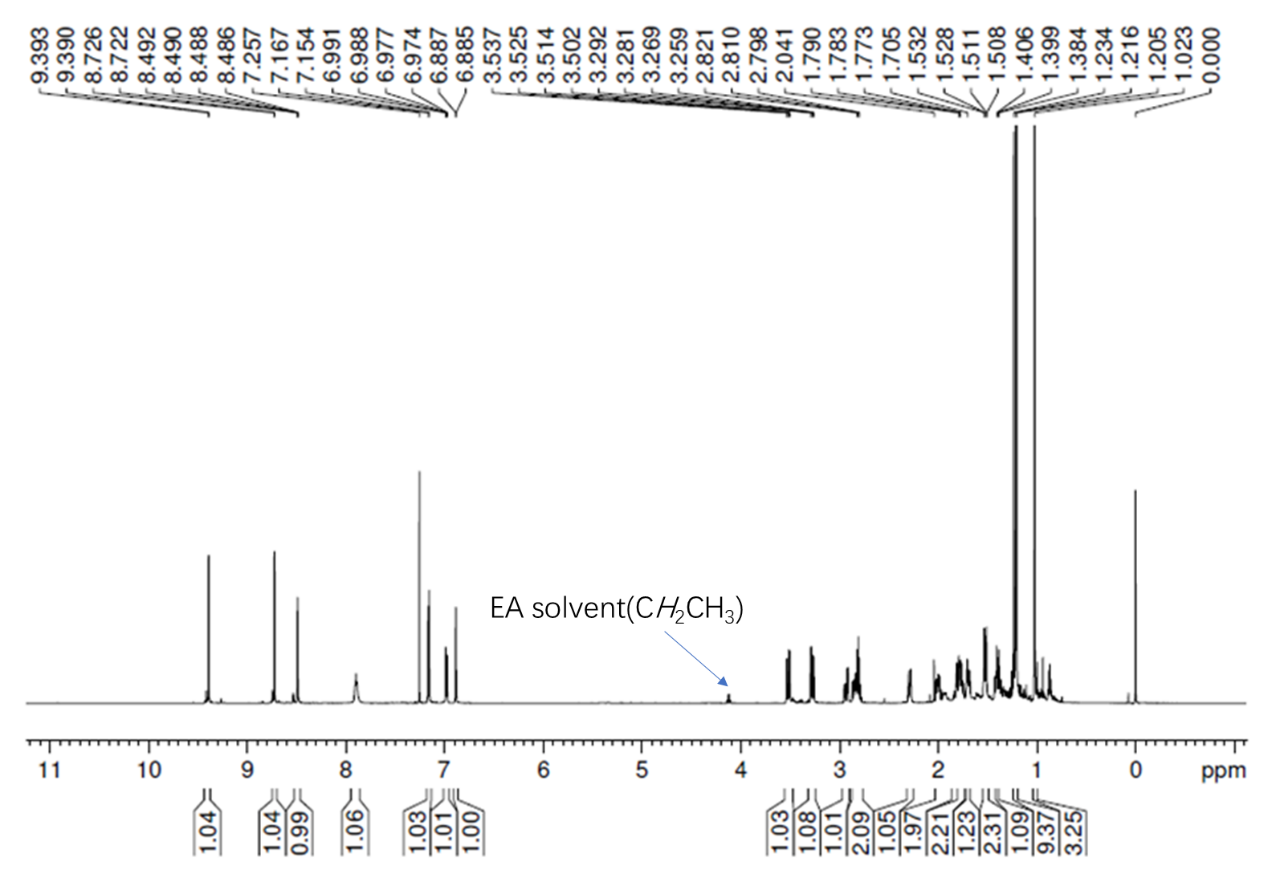
^**

Fig.S13 ^1^H NMR (600 MHz, CDCl_3_) spectrum of compound **L^7^**

**^
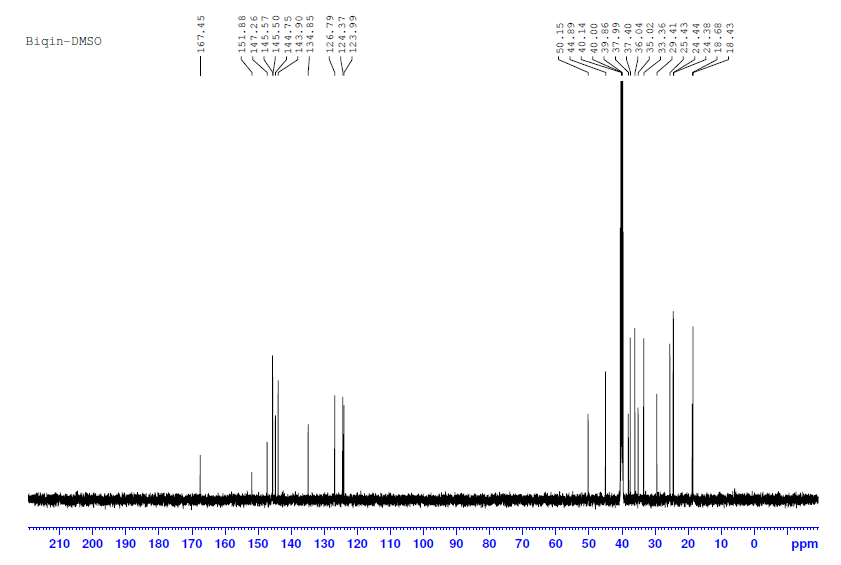
^**

Fig.S14 ^13^C NMR (151 MHz, (CD_3_)_2_SO) spectrum of compound **L^7^**

**^
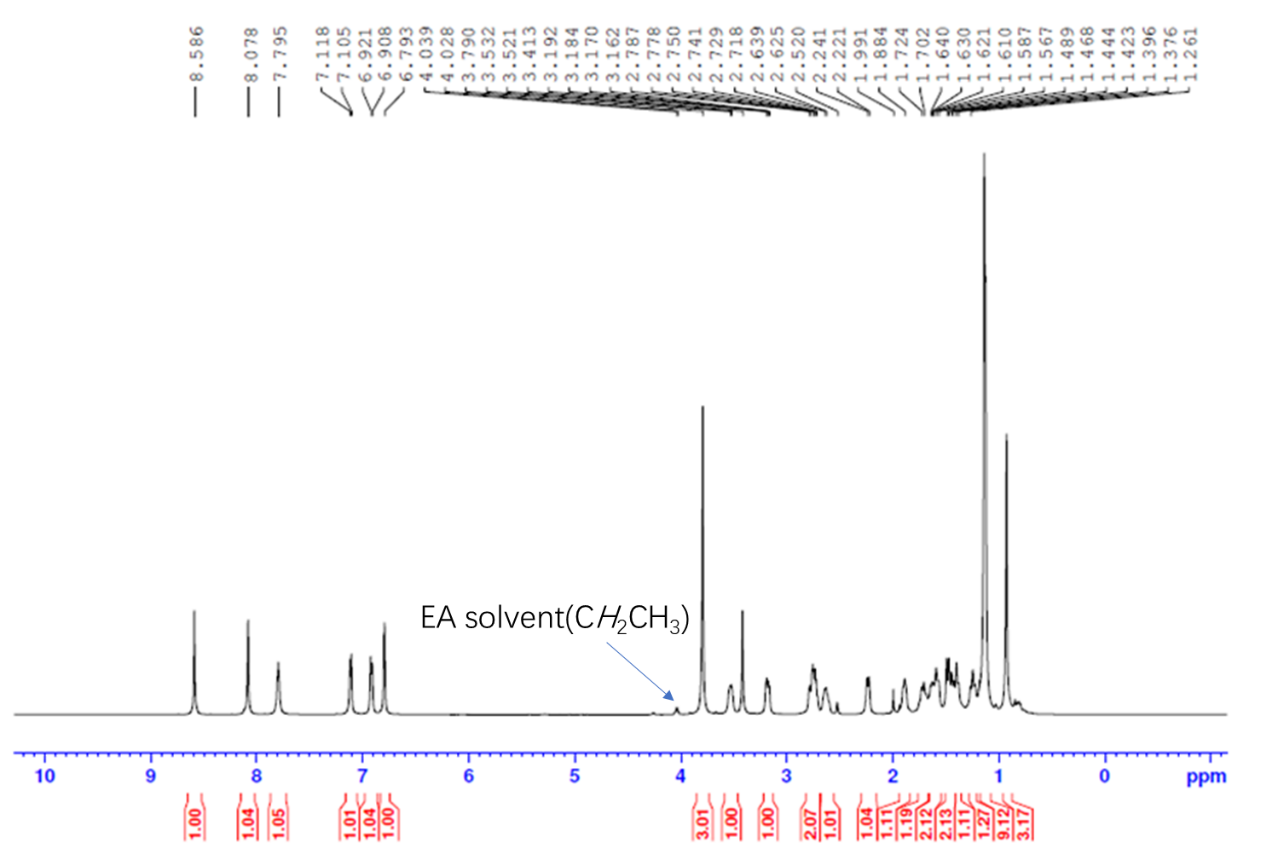
^**

Fig.S15 ^1^H NMR (600 MHz, (CD_3_)_2_SO) spectrum of compound **L^8^**

**^
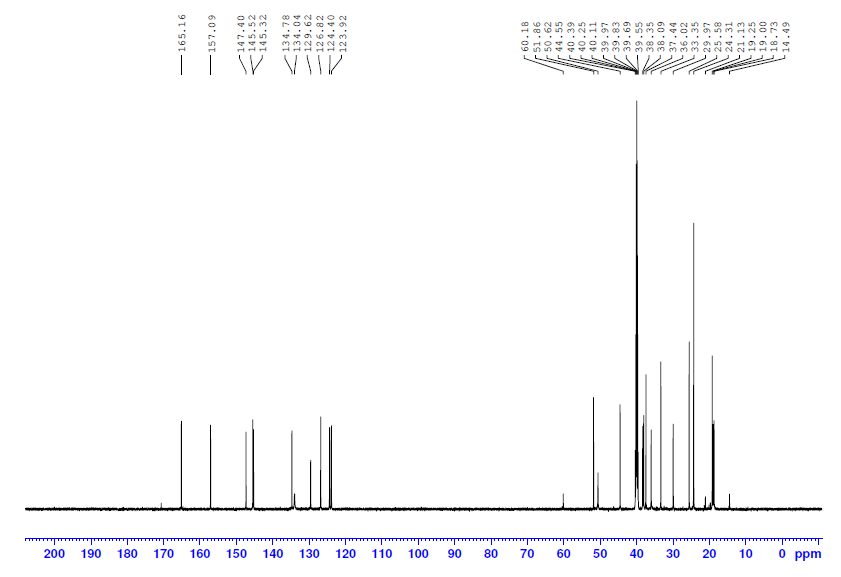
^**

Fig.S16 ^13^C NMR (151 MHz, (CD_3_)_2_SO) spectrum of compound **L^8^**

**^
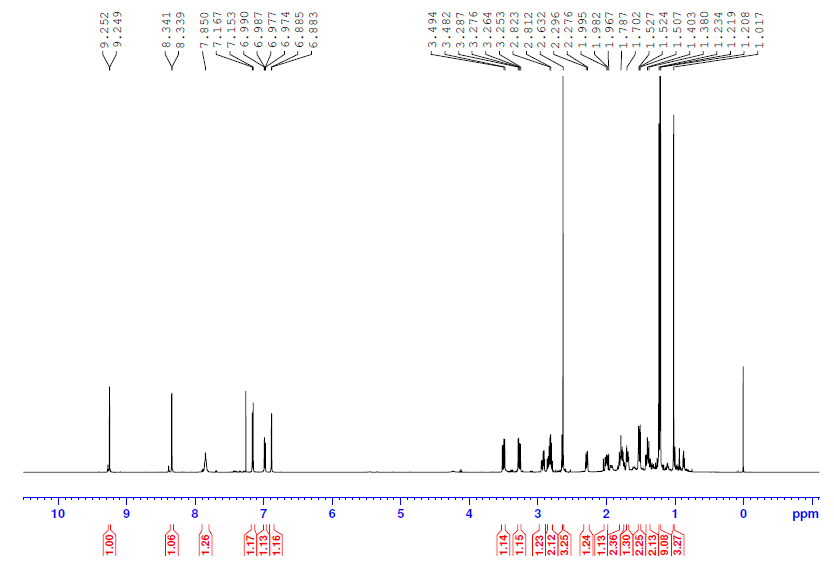
^**

Fig.S17 ^1^H NMR (600 MHz, CDCl_3_) spectrum of compound **L^9^**

**^
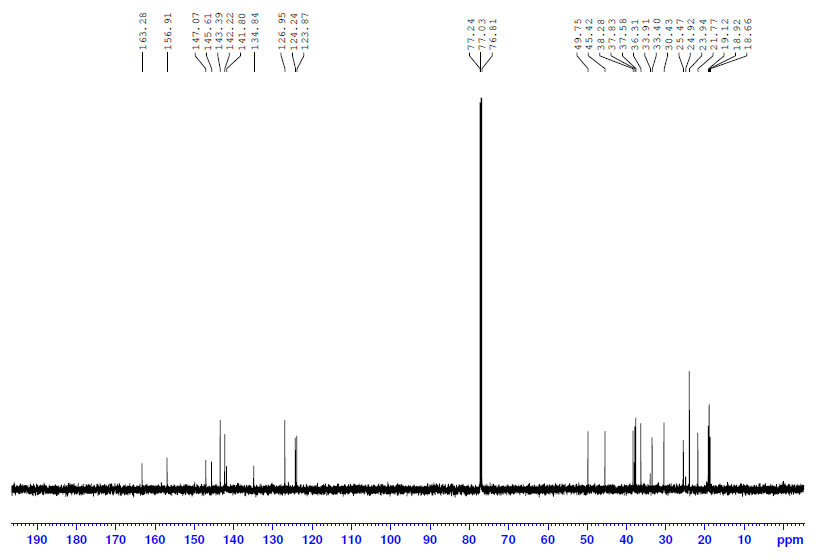
^**

Fig.S18 ^13^C NMR (151 MHz, CDCl_3_) spectrum of compound **L^9^**

**^
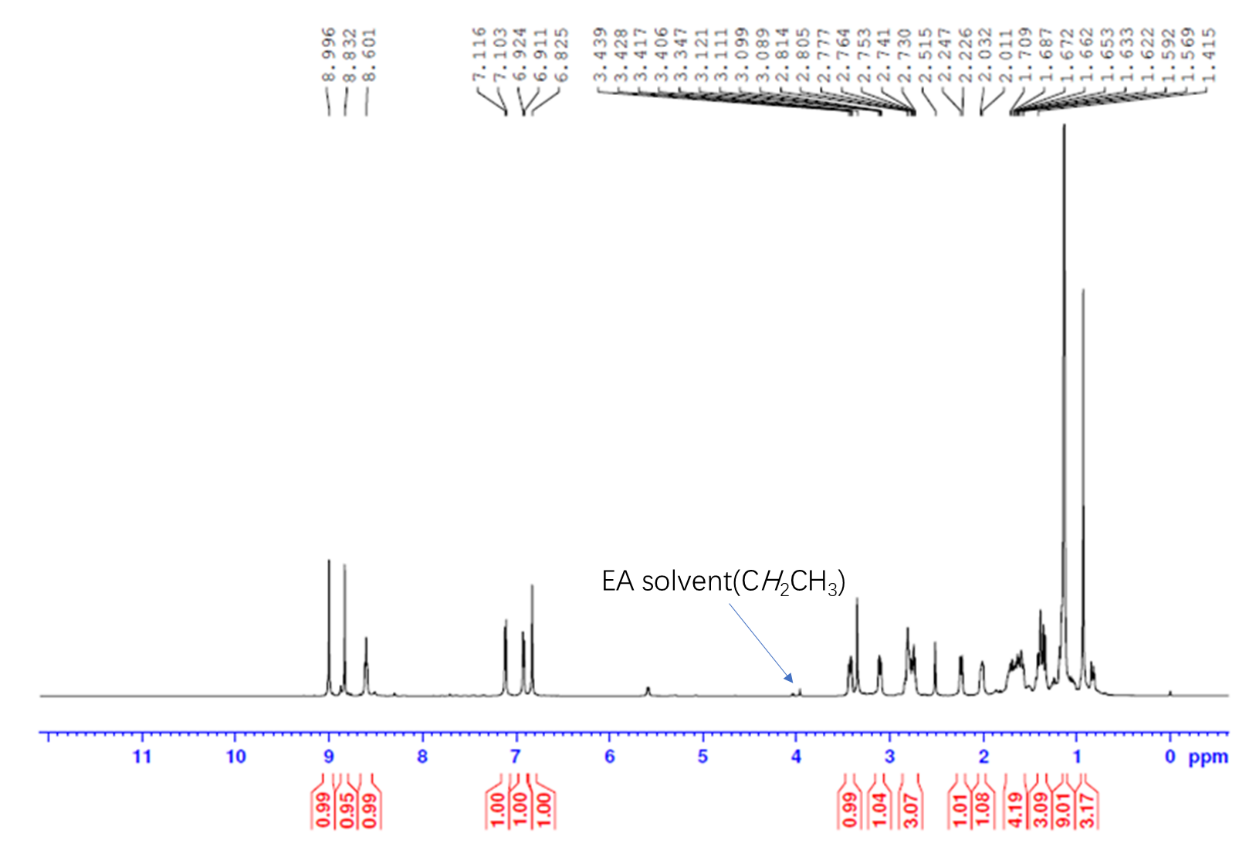
^**

Fig.S19 ^1^H NMR (600 MHz, (CD_3_)_2_SO) spectrum of compound **L^10^**


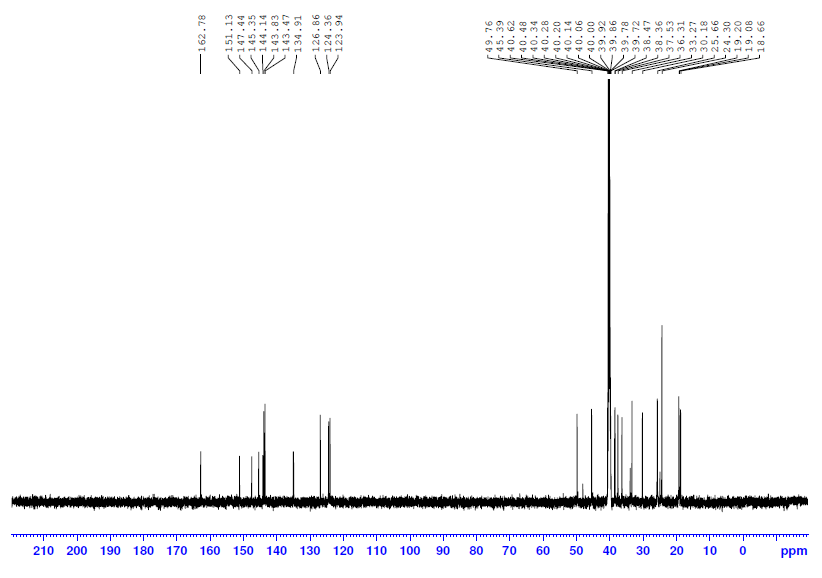


Fig.S20 ^13^C NMR (151 MHz, (CD_3_)_2_SO) spectrum of compound **L^10^**
